# Synergistic integration of extracellular vesicles and metal-organic frameworks: unlocking new opportunities in disease diagnosis and therapy

**DOI:** 10.7150/thno.113474

**Published:** 2025-07-28

**Authors:** Peiye Xu, Jiaxuan He, Ting Xu, Weijie Wang, Baihui Wu, Rongbing Chen, Hanbing Wang, Qinsi Yang, Wei Wu, Da Sun

**Affiliations:** 1 Institute of Life Sciences & Biomedical Collaborative Innovation Center of Zhejiang Province, Wenzhou University, Wenzhou 325035, China.; 2 School of Life and Environmental Science, Wenzhou University, Wenzhou 325035, China.; 3 Department of Biomedical Engineering, City University of Hong Kong, Hong Kong SAR 999077, China.; 4 Department of biotechnology, the University of Hong Kong, Hong Kong SAR 999077, China.; 5 Wenzhou Institute, University of Chinese Academy of Sciences, Wenzhou 325001, China.; 6 Key Laboratory for Biorheological Science and Technology of Ministry of Education, Bioengineering College of Chongqing University, Chongqing 400044, China.; 7 Jin Feng Laboratory, Chongqing, 401329, China.

**Keywords:** metal-organic frameworks, extracellular vesicles, synergistic systems, diagnosis, nanomedicine

## Abstract

The combination of extracellular vesicles (EVs) and metal-organic frameworks (MOFs) is a cutting-edge strategy in nanomedicine, leveraging the immune evasion, targeting capabilities, and biocompatibility of EVs with the high loading capacity and tunable functionality of MOFs. This review comprehensively discusses the latest advancements in the EV-MOF collaborative system, including its combined form and preparation process, focusing on its synergistic applications in disease diagnosis and treatment. EV-MOF platforms have demonstrated enhanced sensitivity in biosensing and bioimaging, offering new avenues for early cancer detection using signal amplification and fluorescence imaging technologies. Therapeutically, EV-MOF systems have demonstrated significant promise in drug delivery, cancer treatment, rheumatoid arthritis, ulcerative colitis, wound healing, bone regeneration, and anti-infection applications, delivering targeted therapies with controlled drug release, and improved biocompatibility. Despite these advancements, challenges remain in optimizing the binding methods between EVs and MOFs, ensuring their stability, and understanding their *in vivo* mechanisms. Addressing these may be key to unlocking the full clinical potential of EV-MOF systems. This review provides a critical analysis of the current state of research, offering insights and guidance for future exploration in this rapidly evolving field.

## Introduction

Metal-organic frameworks (MOFs), a class of hybrid porous materials composed of metal ions or clusters linked by organic ligands, have gained attention since their discovery by Yaghi *et al*. in 1995 1. MOFs are promising candidates for biomedical applications, particularly in disease diagnosis and therapy, due to their unique structural tunability, exceptionally high surface area, and versatile functionality 2. Over the past two decades, advancements in MOF design have enabled the development of multifunctional platforms capable of precise drug delivery, biosensing, and imaging 3. MOFs offer several advantages.

(1) Customizable functionality: The diversity of metal nodes and organic linkers enables the creation of MOFs with tailored properties, including the incorporation of active targeting molecules and stimuli-responsive components 4,5. Attaching targeting molecules such as folic acid to the surface of MOFs enables active targeting of tumor cells 6. This flexibility enables the construction of specialized nanoplatforms that meet specific diagnostic and therapeutic requirements.

(2) Efficient encapsulation and controlled release: Due to their high porosity and large surface area, MOFs can encapsulate numerous therapeutic agents and release them in a controlled manner in response to environmental stimuli, such as pH and enzymes, thereby enhancing therapeutic precision and minimizing off-target effects 7,8.

(3) Biocompatibility and degradability: Many MOFs exhibit high biocompatibility and biodegradability, making them suitable for *in vivo* applications 9. Some zinc(Zn)-based and iron(Fe)-based MOFs can degrade gradually under physiological conditions into low-toxicity metal ions and organic ligands and do not accumulate long-term in the body 10. Green synthetic techniques can be applied to minimize toxicity and ensure safe clearance from the body, thereby enhancing their potential for clinical translation 11.

MOFs demonstrate significant potential; however, they also face challenges, such as limited targeting ability, susceptibility to immune clearance, and suboptimal loading capacity. These limitations highlight the need for hybrid systems with enhanced biofunctional properties.

Extracellular vehicles (EVs), naturally secreted by cells and involved in intercellular communication, provide a complementary solution 12. EVs can encapsulate a diverse array of biomolecules, including proteins, lipids, and nucleic acids, making them versatile carriers for diagnostics and therapeutics 13,14. Moreover, their inherent ability to traverse biological barriers, coupled with their low immunogenicity, makes EVs ideal candidates for enhancing the biocompatibility and targeting precision of nanomaterials 15.

**Figure 1:**
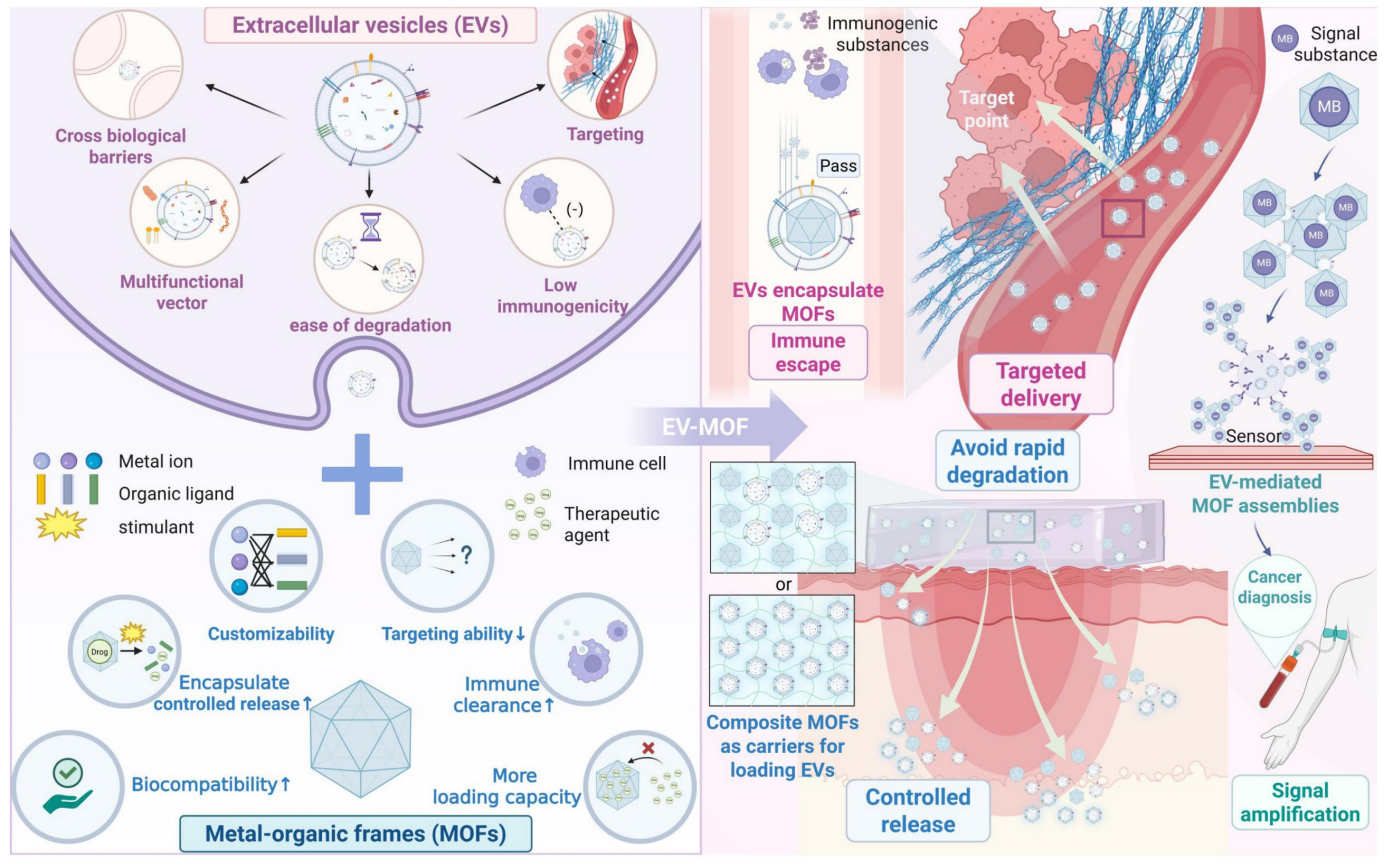
Schematic of EVs, MOFs, and EV-MOF systems. Upper left: EV characteristics include crossing biological barriers, multifunctional vector capability, ease of degradation, low immunogenicity and targeting ability. Lower left: MOF features include relatively high biocompatibility, facile drug encapsulation and controlled release, customizability, low targeting ability, susceptibility to immune clearance, and limited load-bearing capacity. Right: Three types of EV-MOF systems are presented: EVs encapsulate MOFs (pink font), which feature immune escape and targeted delivery; composite MOFs as carriers for loading EVs (blue font), which provide controlled release and avoid rapid degradation; and EV-mediated MOF assemblies (green font), which allow for signal amplification. Created using BioRender.com.

The integration of MOFs with EVs creates a synergistic system that combines diagnostic and therapeutic capabilities. Furthermore, EVs provide immune evasion and targeted delivery, while MOFs contribute to structural integrity and controlled release mechanisms. Connecting the two can also load more signal substances to achieve signal amplification 16. This hybrid system has great potential for addressing key challenges in cancer treatment, tissue repair, regenerative medicine and cancer diagnosis (**Figure 1**).

Despite these promising advances, clinical translation of EV-MOF systems remains in its early stages. In this review, we provide a comprehensive overview of the EV-MOF synergistic system for the first time, focusing on its preparation methods, biomedical applications, and prospects in clinical practice. We aimed to provide a roadmap for the future development of this innovative precision medicine platform by highlighting recent breakthroughs and addressing current challenges.

## 2. Synergistic Effect of EVs with MOFs

The integration of MOFs and EVs has unlocked new possibilities in biomedical applications. MOFs, with their large surface area and tunable porosity, are ideal carriers for drugs and imaging agents; however, they face challenges such as immune clearance and limited targeting 17. EVs, as natural carriers for intercellular communication, offer high biocompatibility and precise targeting; however, their short half-life and lack of controlled release limit their efficacy 18. The combination of MOFs and EVs creates a synergistic platform in which MOFs enhance EV stability and controlled release, while EVs provide immune evasion and tissue-specific targeting. This hybrid system demonstrated superior performance in diagnostics and therapies, particularly in cancer treatment and biosensing.

In this section, we explore the binding mechanisms and preparation techniques of these synergistic systems, highlighting their potential to overcome the limitations of individual components.

### 2.1 The binding form of EVs in collaboration with MOFs

#### 2.1.1 EVs encapsulate MOFs

MOFs exhibit remarkable potential for drug delivery and diagnostic applications due to their high porosity and functional versatility. However, when applied *in vivo*, MOFs often encounter challenges such as the formation of a protein corona, which alters their surface properties and reduces their targeting ability 19,20. Furthermore, immune recognition and subsequent clearance, as well as burst release effects, may limit therapeutic efficacy 21. Surface modification with polyethylene glycol (PEG) and ligands (aptamers, liposomes, and peptides) can partially mitigate these issues. However, they often introduce immunogenic responses, increase production complexity, and raise costs 22,23.

In contrast, encapsulating MOFs in EVs offers several advantages. EVs, naturally derived from cell membranes, possess intrinsic biocompatibility and immune evasion properties 24. Their lipid bilayer structure prevents nanoparticle aggregation and facilitates stable cargo encapsulation (studies have found an encapsulation efficiency of 76.74 ± 6.72% for MOF-loaded EVs) 25, providing a controlled release mechanism across biological barriers 26. Compared to traditional lipid-based carriers, such as liposomes, EVs demonstrate superior immunocompatibility and targeting precision, making them ideal for use in hybrid EV-MOF systems 27.

Furthermore, EVs can better mimic the biological functions of cell membranes without requiring complex preparation processes 28. Unlike cell membrane-coated MOFs, which require multiple steps of mechanical processing to downsize the membranes to fit the nanoscale, EVs are naturally nanosized and can easily encapsulate MOFs without compromising their structural integrity. Moreover, EVs possess inherent targeting abilities, allowing them to pinpoint specific cells or tissues with greater precision than synthetic nanoparticle systems 29.

**Figure 2:**
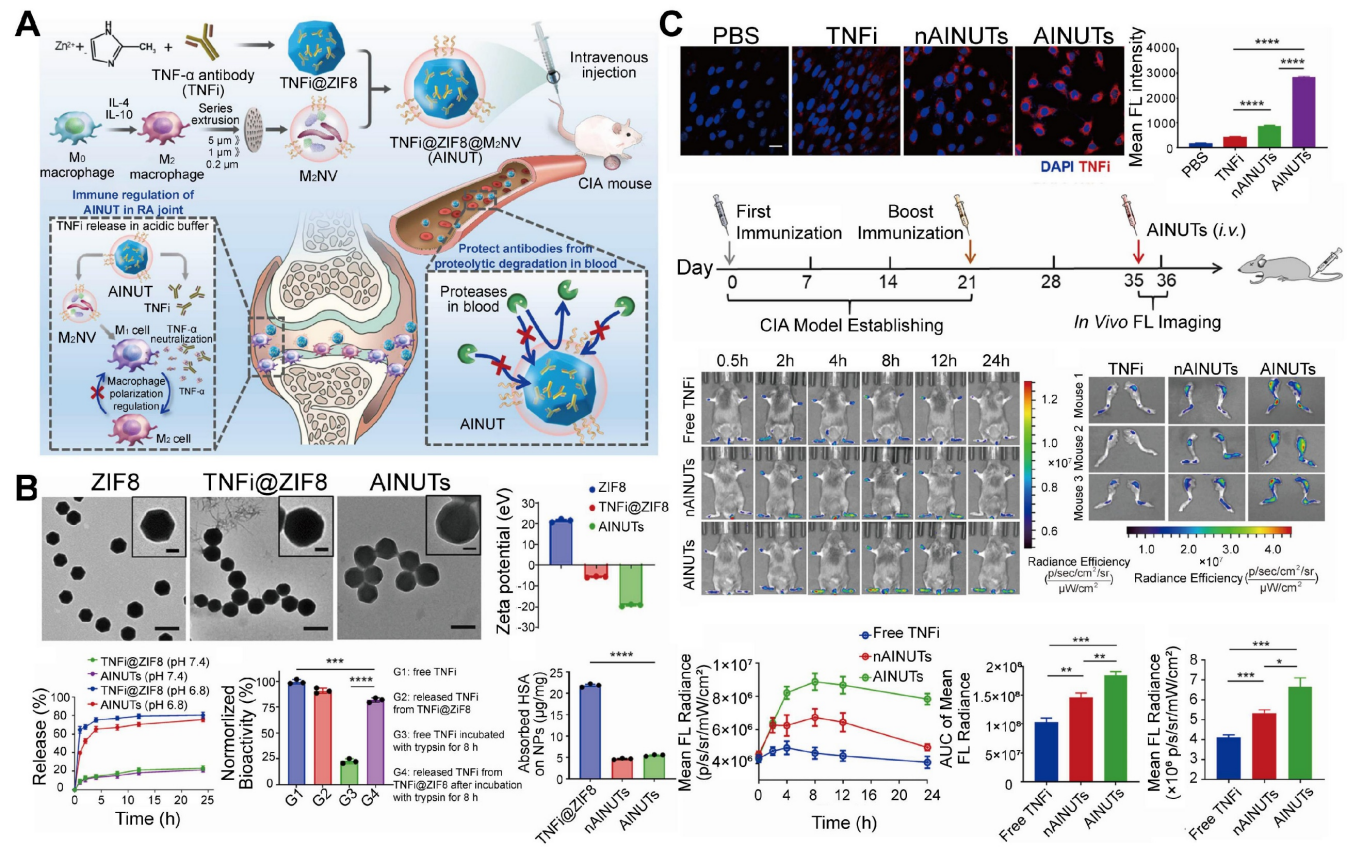
EVs encapsulate MOFs. **A**. Schematic representation of the preparation and anti-inflammatory mechanism of AINUTs, comprising zeolite imidazolate frameworks-8 (ZIF-8) encapsulated with anti-tumor necrosis factor-α (TNF-α) antibody (TNFi) and coated with M_2_ macrophage-derived vesicle (M_2_NV) membrane. **B**. Representative images of transmission electron microscopy (TEM) and zeta potential of ZIF-8, TNFi@ZIF-8, and AINUTs, and different nanoparticles release properties, biological activity, and protein adsorption properties under different conditions. AINUTs and TNFi@ZIF8 can release TNFi on demand in acidic environments. ZIF-8 can protect TNFi from protease degradation and maintain its molecular structure and biological activity. Surface coating of M_2_NVs can significantly reduce non-specific protein adhesion, thereby prolong the circulating half-life and reduce the clearance of the mononuclear phagocytic system (scale bar, 200 nm). The insert depicts a magnified image of a single nanoparticle (scale bar, 50 nm). **C**. Targeting efficiency of AINUTs in rheumatoid arthritis (RA) *in vitro* and *in vivo*. *In vitro*, AINUTs targeted inflammatory HUVECs more effectively, with a stronger binding affinity than TNFi@ZIF8 NPs coated with liposomes (nAINUTs) and native TNFi. *In vivo*, AINUTs exhibited significantly improved accumulation and sustained presence in inflamed joints compared to nAINUTs and TNFi, owing to the high binding affinity of M_2_NVs. Adapted with permission from 34, copyright 2022, Elsevier B.V.

Additionally, some EVs exhibit inherent functions, such as anti-inflammatory and immune response activation, which can synergize with MOF-loaded drugs to achieve powerful therapeutic functions 30,31. Beyond this, EVs can alter the microenvironment at the disease site via mechanisms such as neutralizing toxins and reprogramming the tumor microenvironment (TME) 32,33, thereby accelerating and enhancing the therapeutic effect of MOFs at the disease site. Accordingly, EV-encapsulated MOFs represent a cutting-edge platform that combines "stealth + targeting + protection+ potentiation", significantly improving the biocompatibility, stability, and efficacy of MOF-based nanotherapies. EVs encapsulate MOFs; EVs serve as the outer shell, forming a core-shell MOF@EV structure, including immune nanonuts (AINUTs) (**Figure 2**).

#### 2.1.2 Composite MOFs as carriers for loading EVs

EVs, as a nanomedicine, are widely recognized for their therapeutic potential in tissue repair and regeneration, primarily due to their ability to mediate intercellular communication and deliver bioactive molecules. Simultaneously, EVs, as independent units carrying enzyme activity, are attractive natural biocatalytic platforms 35. However, when applied independently, EVs have significant limitations, including enzyme-loading capacity limited by nanoscale dimensions, rapid degradation *in vivo*, and insufficient accumulation at target sites 36,37. These challenges result in suboptimal therapeutic outcomes, particularly in scenarios requiring prolonged and localized delivery.

To overcome these limitations, MOFs have emerged as promising carriers for stabilizing and sustaining release EVs. MOFs exhibit high porosity, large surface area, and tunable composition, making them ideal candidates for the adsorption and surface mineralization encapsulation of EVs via coordination bonds and electrostatic interactions 38,39. This enables EVs to be incorporated into a controllable release system, enhancing their therapeutic efficiency by protecting them from rapid clearance while facilitating targeted and prolonged release.

In addition to offering a structural platform, MOFs enhance the functionality of EV-based therapies by incorporating metal ions, including zirconium (Zr^4+^), copper (Cu^2+^), and magnesium (Mg^2+^), which provide additional bioactive properties, such as antimicrobial and osteogenic activity 40,41. These metal ions play a critical role in promoting tissue regeneration, particularly in bone healing and wound repair, by supporting cell proliferation, differentiation, and angiogenesis 42. Furthermore, certain light-responsive MOFs can form EV@MOF core-shell structures through biomineralization on EV surfaces, thereby enhancing biocatalytic stability and enabling synergistic effects between photocatalysis and enzymatic catalysis 43. Moreover, the high surface area of MOFs enables adsorption and controlled release of growth factors and other therapeutic agents, further amplifying the regenerative capacity of the composite system 44.

**Figure 3:**
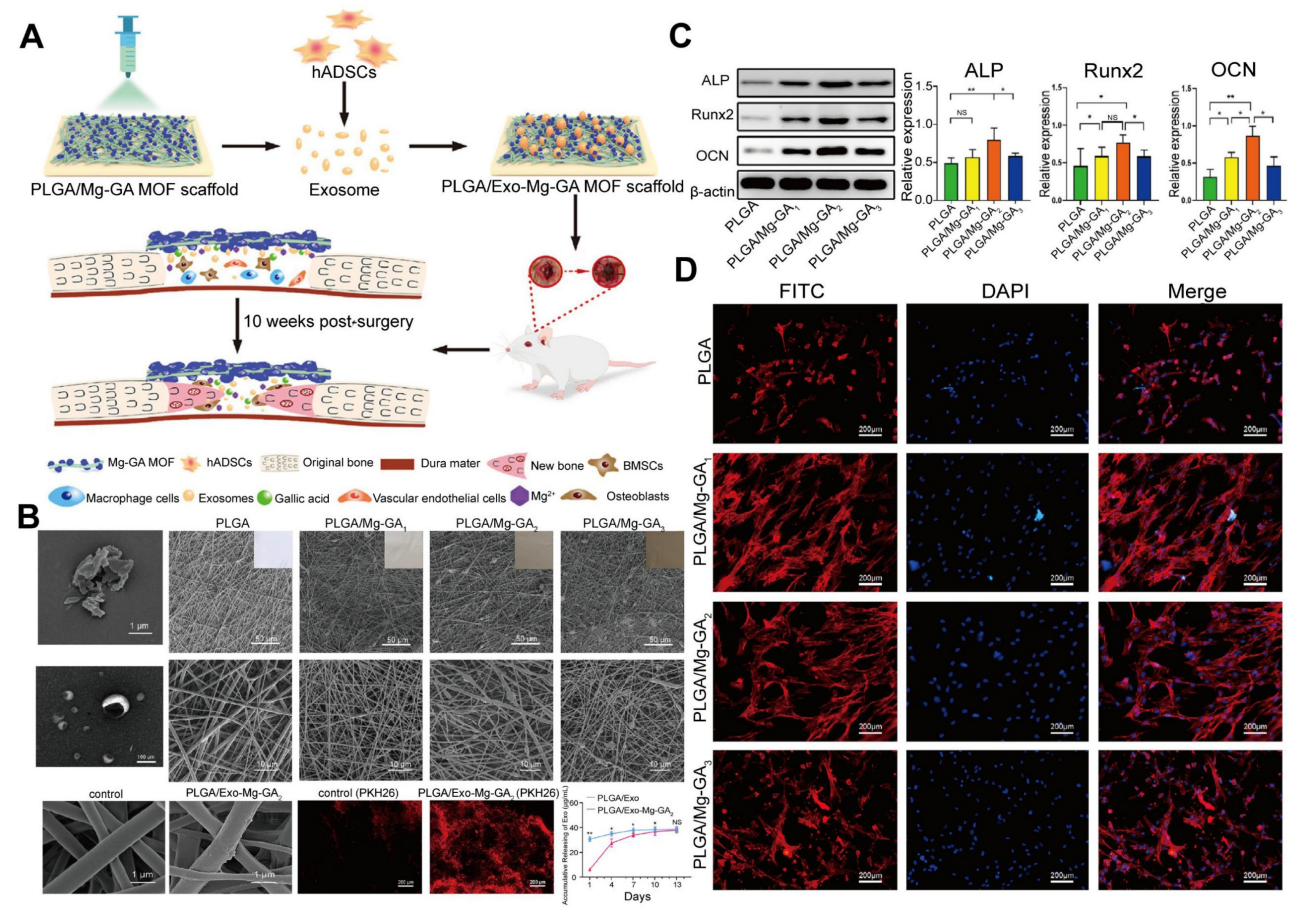
Composite MOFs as carriers for loading EVs. **A**. Schematic representation of the PLGA/Mg-GA MOF composite scaffold loaded with hADSC exosomes for bone tissue regeneration. **B**. Scanning electron microscopy (SEM) and TEM images of the EV-loaded composite scaffold and its components. The addition of Mg-GA MOF particles roughened the PLGA scaffold surface, and the EVs exhibited a disc-shaped double-layer membrane structure. The PLGA/Exo-Mg-GA_2_ scaffold exhibited higher EV attachment and continuous, sustained EV release than the control (PLGA). **C**. Enhanced expression of osteogenic proteins in hBMSCs cultured on PLGA/Exo-Mg-GA_2_ scaffolds. **D**. Immunofluorescence image of hBMSCs on the PLGA/Mg-GA MOF scaffold, showing cells exhibiting shuttle-like and polygonal morphologies, with obvious pseudopodia, along with observable intercellular filaments. Adapted with permission from 47, copyright 2022, Elsevier B.V.

MOFs are versatile because they can be combined with hydrogels, nanofibers, and other biocompatible polymers to form composite materials with improved mechanical properties and biodegradability 45. These hybrid structures create a three-dimensional (3D) matrix that provides mechanical support and mimics the extracellular matrix, promoting cell adhesion and nutrient exchange, thereby creating a more favorable environment for tissue regeneration 46. This composite strategy exhibits unique advantages in payload capacity: Studies have demonstrated that MOF-polymer composites achieve an EV loading efficiency of up to 40 μg/mL, surpassing pure polymer by over two orders of magnitude, while the EV loading of pure polymers is nearly negligible 47. The combination of structural optimization, improved drug delivery performance, and biological synergistic advantages makes EV-MOF composites ideal for applications in tissue engineering and regenerative medicine 48,49. Composite MOFs serve as carriers for loading EVs, either in the form of scaffolds or through encapsulation. For example, a PLGA/Mg-GA MOF composite material scaffold has been used to load exosomes derived from human adipose-derived mesenchymal stem cells (hADSCs) (**Figure 3**).

#### 2.1.3 EV-mediated MOF assemblies

MOFs have emerged as highly effective materials for biosensing applications due to their 3D porous structures, large surface area, and abundance of metal active sites 50-52. These properties enable MOFs to serve as a platform for the development of high-performance biosensors. However, when applied independently, MOFs are limited by the surface area of the electrode interface and the size constraints of individual MOFs, which restrict their ability to capture and detect biomolecules efficiently 53.

The incorporation of EVs into MOF assemblies is a promising solution to these limitations. EVs are naturally rich in hydrophilic phosphate groups on their surface 54, which can form strong coordination bonds with metal ions, Zr^4+^ and titanium (Ti^4+^), found in MOFs 55,56. This metal-phosphate coordination facilitates the assembly of EVs with MOFs, enabling the creation of functional nanomaterials or nanostructures that act as highly efficient signal amplifiers in biosensing. Moreover, the phosphate heads on EVs act as "molecular glue", enabling the assembly of MOFs loading electroactive molecules (methylene blue, MB), into superstructures, with a loading efficiency of 78.7% loading MB into individual MOFs. Experimental studies revealed that increasing the EV-to-Zr-MOF@MB ratio from 1:2 to 1:4 triggers a morphological evolution from discrete Zr-MOF@MB complexes (monomers) to dimeric or trimeric clusters, ultimately forming multi-cluster integrated architectures. This structural progression directly enhances functional performance. Compared to monomeric MOF probes (detection range: 1×10^3^-1×10^8^ particles/mL), the multi-cluster exhibited at least a 10-fold improvement in sensitivity (detection threshold as low as 1×10^2^ particles/mL) while maintaining high specificity. The single-particle level (1 particle per 100 μL sample) exceeded that of most of the reported EV sensors. Additionally, the sensor exhibited a signal difference of up to 11 times when distinguishing between cancerous and benign EVs, indicating excellent specificity. Based on this sensitive and specific strategy, the EV-MOF sensor can analyze EVs for seven cancer types and accurately distinguish between cancer patients and healthy individuals in a clinical cohort 57. This superassembly amplifies the signal detection capabilities of the sensor by increasing the density of MOFs, which capture and identify biomolecules, thereby improving the sensitivity and accuracy of the biosensing platform.

**Figure 4:**
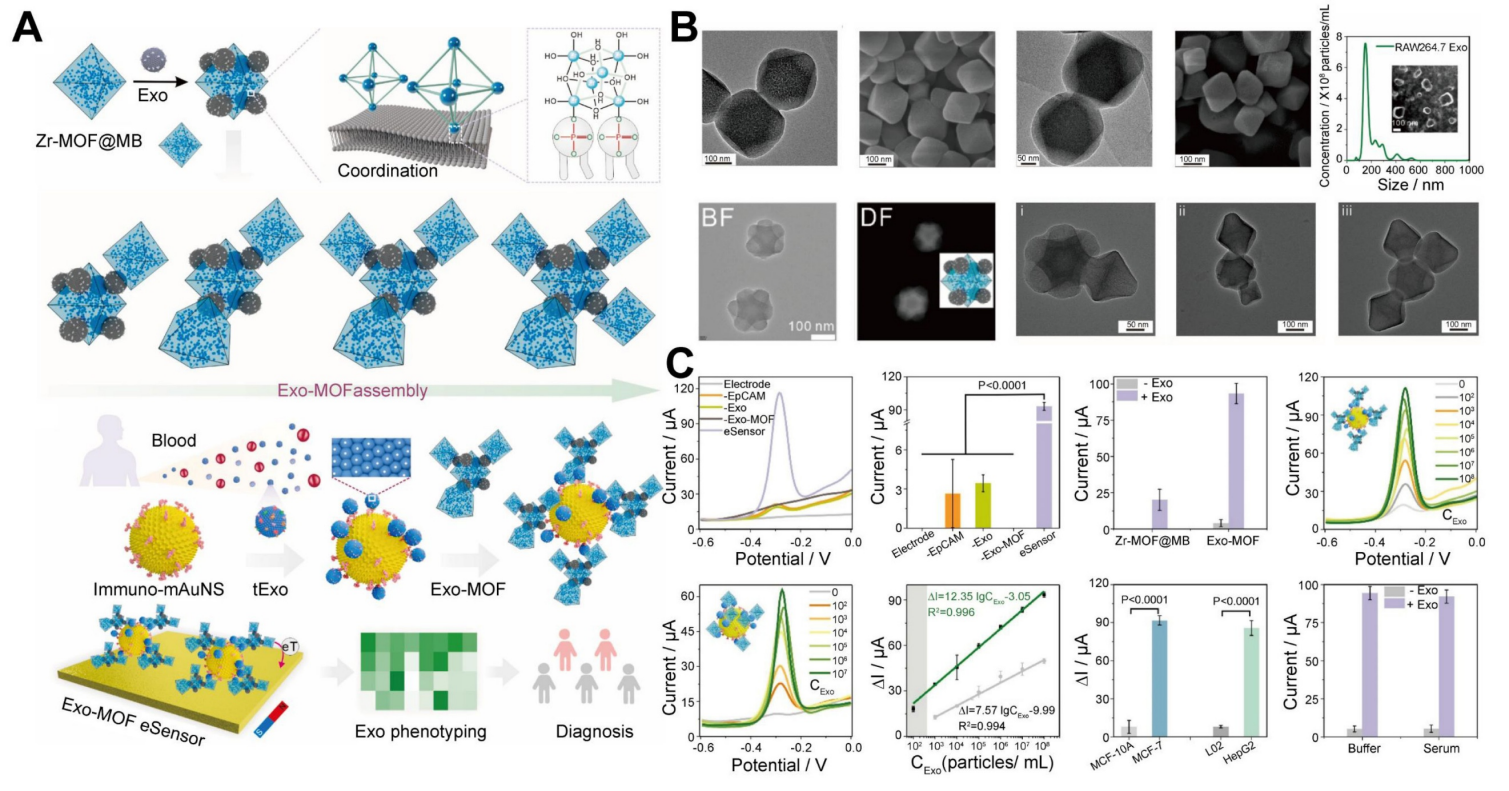
EV-mediated MOFs assemblies. **A**. Schematic of the Exo-MOF eSensor for exosome analysis and cancer diagnosis, highlighting the Zr-phosphate coordination-driven assembly of the Exo-MOF signal amplifier and its coupling to immunomagnetic gold (Au) nanostars. **B**. SEM, TEM, and nanoparticle tracking analysis (NTA) images of Zr-MOF, Zr-MOF@MB, Exo, Zr-MOF@Exo core-satellite structure, and the Exo-MOF super redox signal amplifier, illustrate the formation of a Zr-MOF@Exo nanostructure as Exo assembles on the Zr-MOF octahedron. The morphological transitions with increasing Exo/Zr-MOF@MB ratio, resulted in the formation of a super redox signal amplifier. **C**. The Exo-MOF eSensor demonstrates ultra-sensitive and specific detection of tExo, with enhanced signal strength, signal gain, and broader dynamic range compared to other systems, along with excellent specificity and anti-interference capabilities. Adapted with permission from 57, copyright 2024, Elsevier B.V.

This superassembly approach, known as EV-mediated MOF assemblies (where EVs serve as molecular glue facilitating MOF assemblies), represents a significant advancement in biosensor design, allowing for more efficient and robust detection of biomolecules in complex biological environments (**Figure 4**). This system provides an innovative platform that leverages the natural properties of EVs and the structural flexibility of MOFs to enhance the performance of biosensors in medical diagnostics and other applications.

#### 2.1.4 Comparison with other organic or inorganic nanocarriers

Various delivery systems, including liposomes, polymer nanoparticles, and inorganic nanomaterials have recently emerged as promising drug carriers in nanomedicine. These delivery systems provide several advantages, including mature production processes and high yields 58. The United States Food and Drug Administration (FDA) has approved some nanocarriers for use in clinical practice 59. However, each system has limitations; for example, liposomes are prone to leakage, polymer nanoparticles have insufficient drug loading, and inorganic materials have poor biocompatibility 60,61. The EV-MOF system, is a "biosynthetic" hybrid system that combines the natural biological activity of EVs with the structural tunability of MOFs, endowing it with unique performance advantages (**Table 1**).

Regarding drug loading efficiency, the EV-MOF system leverages the high porosity characteristics of MOFs, enabling them to capture active molecules and drugs. Some inorganic nanomaterials, such as mesoporous silica, have larger pore sizes (2-50 nm), increasing their loading capacity. The drug loading capacity of polymer nanoparticles achieved via adsorption or coupling is limited 62. Traditional liposomes use phospholipid membranes to encapsulate lipophilic drugs or water nuclei to load hydrophilic drugs; however, drug retention is insufficient 63.

Regarding *in vivo* stability, the MOFs of the EV-MOF system are easily recognized and removed by the immune system 64. However, the natural phospholipid bilayer membrane of EVs resists enzymatic hydrolysis, thereby prolonging their circulating half-life 65,66. Traditional liposomes are easily adsorbed by serum proteins, triggering complement activation and leading to rapid clearance. PEG modification can extend the half-life 67. Polymer materials such as PLGA can slowly release drugs via hydrolysis 68. Silica and Au nanoparticles degrade slowly in the body or hardly degrade at all, and are chemically inert 69. The stability of the carrier must maintain a delicate balance with its biocompatibility. Excessive stability can easily lead to long-term retention of the carrier in the body and cause toxicity 70.

From the immunogenicity perspective, EVs can escape immune clearance through the surface CD47 protein. Although synthetic liposomes can reduce immunogenicity through surface modification, such as PEGylation, repeated administration of PEGylated liposomes may induce the production of anti-PEG IgM antibodies and complement activation, thereby accelerating blood clearance 71. Most polymers exhibit excellent immunogenicity, but hydrophobic polymers, such as polycaprolactone, may trigger stronger immune responses due to hydrophobic interactions (increased cytokine levels) 72. Some inorganic nanomaterials directly induce immune responses; although their immunomodulatory properties offer potential for developing new therapies in fields such as oncology, excessive immune reactions can directly lead to toxicity 73.

The EV-MOF system inherits the intrinsic homing capacity of EVs while leveraging the tunable size of MOFs to achieve tumor accumulation through the enhanced permeability and retention (EPR) effect, thereby establishing a "dual-targeting" mechanism 74,75. In contrast, conventional drug carriers, such as liposomes, without ligand modification, predominantly rely on passive targeting, including the EPR effect, and exhibit limited efficacy in complex physiological environments 76.

Although direct comparative data remain to be systematically validated, current studies demonstrate that the modular design of the EV-MOF system enables the simultaneous achievement of both high drug loading capacity in MOFs and EV-mediated targeting capabilities, significantly surpassing the single-function limitations of conventional carriers 77. Their biomimetic membrane structure confers prolonged circulation with an extended half-life compared to PEGylated liposomes and immune evasion via EV surface proteins, such as CD47 71. Furthermore, the nanoscale size of EV-MOF, combined with the pH-responsive degradation of MOFs, enhances tumor penetration and enables spatiotemporally controlled drug release 78. Future studies should prioritize comparative experiments to quantitatively assess these advantages. However, the "biosynthetic synergy" strategy of EV-MOF has already established a novel paradigm for addressing critical challenges in nanomedicine, including drug loading efficiency, immune clearance, and tissue distribution.

### 2.2 Preparation of EVs synergistic MOFs

**Figure 5:**
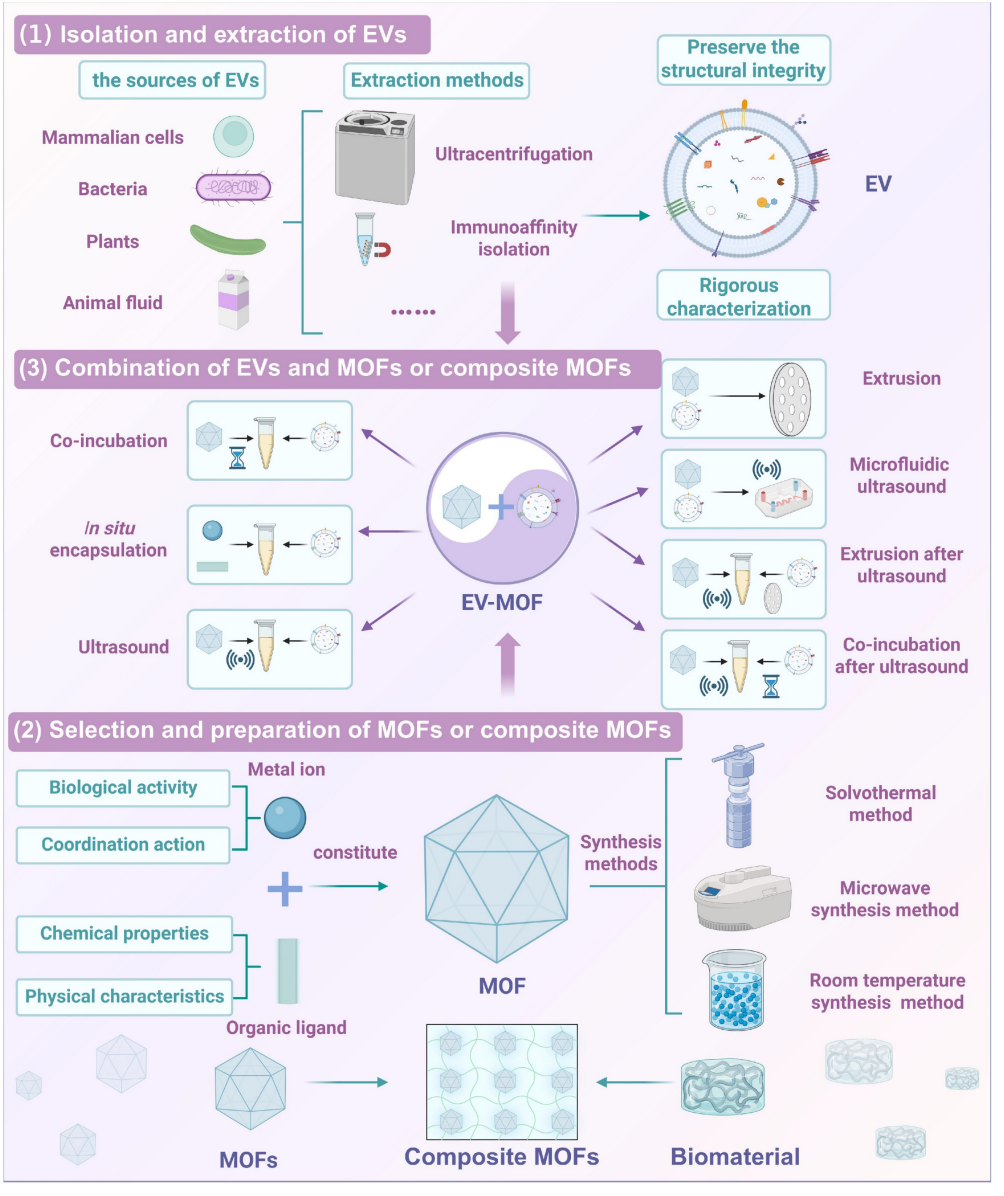
Schematic of the preparation of EVs synergistic MOFs. Top: (1) Isolation and extraction of EVs: including the source of EVs, extraction methods, and obtained EVs must maintain structural integrity and undergo rigorous characterization. Bottom: (2) Selection and preparation of MOFs or composite MOFs: MOFs are formed by metal ions with biological activity and coordination action, and organic ligands with chemical properties and physical characteristics. The preparation methods include solvothermal synthesis, etc. MOFs can be combined with biomaterials to form composite MOFs. Middle: (3) Combination of EVs and MOFs or composite MOFs: describes seven methods for combining EVs with MOFs or composite MOFs. Created using BioRender.com.

EV-MOF preparation involves three critical steps (**Figure 5**) 79. First, EVs must be carefully extracted and purified to retain their biological functions. Second, the synthesis of MOFs is tailored to meet biomedical requirements by optimizing factors, including size, porosity, and surface chemistry. Third, the fusion of EVs with MOFs ensures structural stability and functionality typically achieved via coordination bonds or electrostatic interactions. These steps collectively determine the efficiency and effectiveness of the resulting EV-MOF system, which is vital for applications in drug delivery and biosensing.

#### 2.2.1 Isolation and extraction of EVs

Isolation and extraction of EVs are critical steps in the development of EV-MOF systems because the integrity and functionality of EVs directly affect their therapeutic efficacy. EVs are typically derived from mammalian cells, bacteria, plants, and animal fluids are commonly extracted from cell culture media, bacterial media, plant juice or animal fluids 80-82.

Ultracentrifugation is the most commonly used method for EV isolation because it can efficiently separate EVs based on their size and density 83. Moreover, other classical EVs separation methods based on the physical and biomolecular properties of EVs, including size exclusion chromatography, ultrafiltration, polymer precipitation, and immunoaffinity isolation have been developed to improve purity and yield 84. Beyond classical techniques, several advanced EV isolation strategies have recently emerged, marrying functional specificity to high efficiency. MOF-based capture platforms leverage the high surface area and tunable porosity of MOFs to selectively bind EVs through surface chemistry modulation. This approach enables simultaneous isolation and high-throughput analysis, making it particularly advantageous for biosensor integration and microvolume diagnostics 85. Meanwhile, molecular imprinting technologies have enabled the fabrication of synthetic recognition sites that mimic natural EV surface markers. These platforms demonstrate high selectivity, operational stability, and reusability, demonstrating potential for high-throughput EV screening and enrichment 86. Another innovative approach utilizes supramolecular chemical probes based on host-guest interactions. These probes offer reversible and tunable binding dynamics, facilitating controlled EVs capture and release. This feature renders such systems promising tools for downstream EV proteomics or transcriptomics analysis 87. Although these emerging techniques have yet to see widespread adoption in clinical workflows, their modularity and design flexibility demonstrate strong potential for integration into future diagnostic and therapeutic systems.

Preserving the structural integrity of EVs is particularly important for their immune escape and targeting properties. It ensures the presence of key membrane proteins such as CD47 ("do not eat me" signal) and tetraspanins, which are vital for immune evasion and targeted delivery 88,89.

Furthermore, rigorous characterization of isolated EVs is essential. Some commonly used methods include TEM and SEM to resolve the size and goblet morphology of EVs, NTA and dynamic light scattering (DLS) to analyze the particle size distribution of EVs, and Western blotting (WB) and proteomics to detect proteins on the surface of EVs 90.

#### 2.2.2 Selection and preparation of MOFs or composite MOFs

The design and synthesis of MOFs for biomedical applications requires careful consideration of multiple factors, including metal ion selection, structural characteristics, and biocompatibility. MOFs typically consist of metal nodes linked by organic ligands, forming highly porous structures ideal for drug delivery, tissue regeneration, and diagnostic applications 91-93.

The choice of metal ions is important because it determines the biological activity and functionality of the framework. Metal ions including Zr^2+^, Cu^2+^, and Zn^2+^, provide structural stability and confer additional therapeutic properties, including antimicrobial or osteogenic activity. These ions can interact with EVs via coordination bonds, enhancing the ability of MOFs to encapsulate and deliver bioactive molecules 94.

The organic ligands in MOFs determine their specific surface area, pore size, and chemical properties, directly influencing their capacity to carry therapeutic agents or diagnostic probes 95. Additionally, the selection of special organic ligands can confer MOF with unique properties, including light, electricity, and sound, allowing them to be directly used as therapeutic agents 96,97. To ensure safety and efficiency, MOFs are frequently synthesized using solvothermal or microwave methods, while green solvents, such as water and ethanol, are increasingly used to improve biocompatibility 98. Some MOFs, such as ZIF-8, can be synthesized at room temperature, offering a simpler and more sustainable production route 99.

For composite materials, integrating MOFs with biomaterials, such as hydrogels or nanofibers, enhances mechanical properties, improves biocompatibility, and promotes tissue regeneration. The porous structure of MOFs facilitates cell infiltration and growth, while their controlled degradation releases metal ions that support the repair of damaged tissues 100. This combination creates multifunctional scaffolds that are ideal for use in tissue engineering and regenerative medicine applications.

#### 2.2.3 Combination of EVs and MOFs or composite MOFs

The combination of EVs and MOFs creates a synergistic system that enhances the structural stability and functional efficacy of the resulting nanostructures. Several methods have been developed to integrate these two components, each offering unique advantages and challenges.

(1) Co-incubation: This method involves incubating EVs with MOFs, to allow them to interact and form composite structures. Although this method is simple and protects the structural integrity of EVs, it frequently yields low loading efficiency 101.

(2) *In situ* encapsulation: EVs are mixed with MOF precursors to form MOFs on the EV surface *in situ*, thereby encapsulating the EVs. This one-step approach enhances preparation efficiency, but may have an impact on the active material of EVs 39.

(3) Ultrasound: EV membranes (EM) are transiently disrupted by applying ultrasonic energy, enabling the entry of MOFs to form core-shell structures. This approach improves the loading efficiency but may compromise the stability of EVs 102.

(4) Extrusion: When EVs and MOFs repeatedly pass through porous membranes, mechanical disruption occurs, causing the EVs to reassemble around the MOFs. This method improves the loading efficiency but risks damaging EVs 103.

(5) Microfluidic ultrasound: In this advanced method, EVs and MOFs flow through a microfluidic device under acoustic pressure, thereby facilitating efficient fusion. This approach is fast, scalable, and conducive to mass production 104.

Additionally, hybrid methods such as co-incubation after ultrasound and extrusion after ultrasound, combine the strengths of multiple techniques 25,105, further optimizing loading efficiency while balancing the preservation of EVs structural integrity. Several methods for combining EVs with MOFs or composite MOFs are summarized in **Table 2**.

## 3. Application of the MOF and EV Collaborative System in Disease Diagnosis

The EV-MOF collaborative system offers a promising solution for early and accurate disease diagnosis by combining the immune evasion and targeting capabilities of EVs with the high surface area and tunable chemistry of MOFs. This synergy enhances the sensitivity and specificity of biosensors and bioimaging platforms, enabling the more precise detection of disease biomarkers and improving diagnostic outcomes.

### 3.1 Biological sensing

The accurate and efficient detection of biological substances is a critical challenge in disease diagnosis 107. MOFs, with their large surface area and tunable pore structure, provide an ideal platform for biosensing, but their individual efficiencies are limited 108,109. The limitations hindering MOFs in biosensors can be addressed by assembling individual MOFs into MOF clusters and using EVs as molecular glue for MOFs.

Recent studies have demonstrated that many tumor-derived EV, serving as natural biomarkers, their surface proteins are closely related to carcinogenic mechanisms. These EVs can be non-invasively obtained from body fluids, dynamically reflect disease progression, and are ideal sensing targets for cancer diagnostics 110; however, circulating tumor EVs still face significant challenges in clinical applications. The low abundance of EVs in body fluids of early cancer patients 111, their wide distribution across heterogeneous sizes (30 nm to 10 μm) 112, complex molecular composition and the high dispersion in biological fluids make it challenging to achieve efficient isolation and detection using conventional methods 113.

The integration of EVs into MOF-based sensors to form a new generation of signal amplifiers can enhance sensitivity and specificity. Qin *et al*. 57 developed a superassembly strategy to coordinate EVs with MOFs, optimizing MOF-based signal amplification for ultrasensitive detection of tumor-derived exosomes (tExo). They assembled EV-MOF clusters to significantly amplify electrochemical signals using specific interaction recognition between Zr^4+^ ions in Zr-MOFs and phosphate groups in cancer exosomes. The inclusion of MB in the Zr-MOFs further enhanced the signal strength. To boost signal gain, magnetic nanoparticles with Au nanostars (mAuNSs) functionalized with antibodies were synthesized to capture exosomes more efficiently. These mAuNSs enhanced exosome capture by increasing their interaction with the electrode surface. The captured tExo contains phosphate groups that coordinate with EV-MOF, amplifying the signal and improving sensitivity. This EV-MOF sensor allows for precise tExo quantification, effectively distinguishing patients with breast cancer from healthy individuals and highlighting its potential for early cancer diagnosis.

### 3.2 Biological imaging

Advances in medical imaging technologies, including X-rays, computed tomography (CT), magnetic resonance imaging (MRI), and photoacoustic imaging, have contributed to enhancing disease diagnosis and monitoring 114,115. Biological imaging enables the real-time visualization of physiological and pathological processes, making it critical for early diagnosis and treatment 116. Fluorescence imaging stands out for its non-invasiveness, high sensitivity, and ease of operation 117. MOFs, with their customizable emission properties, have emerged as promising materials for bioimaging due to their ability to encapsulate fluorescent dyes and their high tunability 118,119. MOF-based biosensors have significantly contributed to disease research and diagnosis, especially when combined with EVs, which enhance targeting and immune evasion.

Adenosine triphosphate (ATP), the primary energy source for cellular processes, is a critical marker of cell function, viability, and metabolism, and its fluctuations are closely linked to various diseases 120,121. The sensitive detection of intracellular ATP is essential for biochemical research and clinical diagnosis. MOF-based sensors have demonstrated exceptional capabilities for ATP detection 122, particularly in live cells. EV-coated MOFs have been applied for this purpose. Lv *et al*. 123 developed a microfluidic sonication method to create EM-coated ZIF-8 nanoparticles that demonstrated enhanced phagocytosis evasion compared to uncoated ZIF-8 nanoparticles. Additionally, *in situ* imaging of ATP with EM-ZIF-8 loaded with rhodamine B (RhB) revealed a unique mechanism: ATP disrupts the ZIF-8 structure via competitive coordination with Zn^2+^ ions, thereby restoring the RhB fluorescence. This ATP-triggered fluorescence recovery demonstrates that biomimetic EM-ZIF-8 nanoparticles are a promising platform for intracellular drug delivery and real-time ATP sensing in live cancer cells.

## 4. Application of the MOF and EV collaborative System in Disease Treatment

EV-coated MOFs offer a novel approach to overcoming the limitations of traditional nanotherapy, including poor targeting and immune clearance. EVs enhance biocompatibility and immune evasion, while MOFs provide efficient drug loading and controlled release. This EV-MOF system improves therapeutic efficacy and reduces toxicity by delivering drugs more precisely to target cells. Additionally, MOFs stabilize EVs via coordination bonds and electrostatic interactions, prolonging their therapeutic effects. Simultaneously, MOF + EV produced a 1 + 1> 2 effect. This synergistic system offers precise drug targeting, enhanced biocompatibility, and controlled release, making it a promising strategy for more effective and safe treatments.

### 4.1 Drug delivery

Nanomedicine has become a pivotal approach in modern biomedical research, offering new avenues for treating various diseases via targeted and controlled drug delivery 124. EVs, with their intrinsic role in intercellular communication, have garnered attention as promising therapeutic carriers, particularly in treating cancer and cardiovascular disease 125. Additionally, conventional nanoparticles, such as liposomes, have been extensively employed in clinical settings. However, the effective delivery of these agents remains a persistent challenge 126. Current nanocarriers, such as liposomes and exosomes are frequently cleared rapidly after systemic administration and struggle to maintain effective concentrations at target sites, potentially limiting their therapeutic efficacy 127,128.

To address these limitations, advanced nanocarrier designs that can stabilize and protect therapeutic agents are crucial. An innovative approach by Li *et al*. 129 used nanogrid particles (NGPs), which integrate supramolecular carriers capable of encapsulating larger nanoparticles, including liposomes and exosomes. The NGP system uses cyclodextrin-based MOFs (CD-MOFs) crosslinked with boron linkers, leveraging the high porosity and biocompatibility of CD-MOFs, along with the dual pH and H_2_O_2_ responsiveness provided by boron. This design significantly enhances the distribution and retention of liposomes in lung tissues, while the encapsulated exosomes exhibit protective and sustained-release properties, offering a multifaceted platform for the effective delivery of numerous therapeutic molecules and nanoparticles.

### 4.2 Cancer treatment

Cancer is a leading cause of morbidity and mortality globally 130. Although chemotherapy, protein therapy, gene therapy, immunization therapy and sonodynamic therapy have made significant progress, they still face challenges, including poor targeting, immune clearance, and side effects. Chemotherapy is widely used, but has low delivery efficiency and systemic toxicity 131. Similarly, barriers to protein, immunization and gene therapies include low cellular uptake, enzymatic degradation, and insufficient tumor penetration 132. Gene therapy nucleic acid drugs can specifically act on target cells at the gene level. However, naked nucleic acid drugs do not achieve desired results when administered directly due to their non-specific biodistribution, low cellular uptake, rapid clearance, and enzymatic degradation 133. Despite encouraging progress in sonodynamic therapy, several challenges remain in its clinical application, including insufficient tumor accumulation, high tumor heat tolerance, and distant metastasis, which impede effective tumor treatment 134. As a cutting-edge platform for cancer treatment, EV-MOF demonstrates significant potential to improve the efficacy of current therapies, offering a more targeted and biocompatible approach.

#### 4.2.1 Chemotherapy

**Figure 6:**
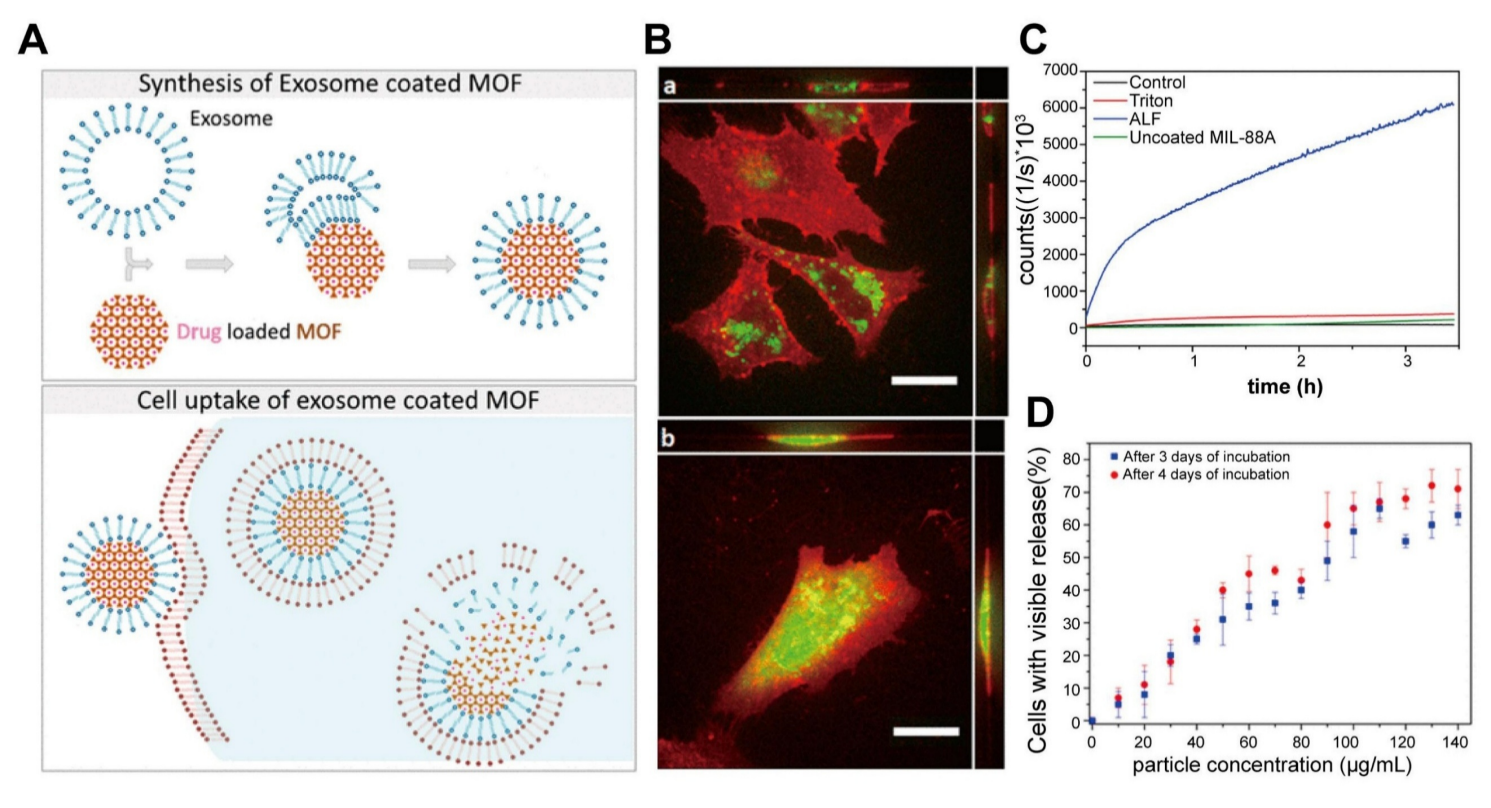
Chemotherapy**. A**. Schematic illustration of exosome-coated MOF synthesis, cellular uptake, and cargo release mechanisms. **B**. Uptake and release of exosome-coated calcein-laden MOF NPs (green) in HeLa cells (red). After two days (a), the particles were internalized with no release. Calcein was released into cells after four days (b) (scale bar, 20 μm). **C**. Fluorescence release assay of calcein from exosome-coated MOF nanoparticles in different solutions. No release in water was indicated, suggesting a tight exosome coating, the uncoated particles leaked steadily into the water. Calcein was released after Triton X-100 treatment. Fluorescence increases in artificial lysosomal fluid (ALF), suggesting MOF breakdown and exosome rupture. **D**. Intracellular calcein release efficiency after incubation with exosome-coated MOF at varying concentrations. Higher nanoparticle concentrations were correlated with increased calcein release. No significant differences were observed between days three and four of incubation. Adapted with permission from 135, copyright 2017, American Chemical Society.

Traditional chemotherapy often faces challenges such as low targeting efficiency, immune clearance, and systemic toxicity. Bernhard Illes *et al*. 135 first proposed a promising solution: EV-coated MOF nanoparticles, as an intelligent and efficient drug delivery system with an "on-board trigger" (**Figure 6**). This system combines the chemically tunable properties of MOF nanoparticles with the biocompatibility and targeting capabilities of EVs to create an innovative drug delivery platform with efficient loading and minimal premature leakage. In this study, MIL-88A nanoparticles, composed of Fe and fumaric acid, were synthesized using the microwave method and loaded with chemotherapy drugs. 3-(4,5)-dimethylthiahiazo(-z-y1)-3,5-di-phenytetrazoliumromide (MTT) cytotoxicity experiments revealed that MIL-88A exhibited good biocompatibility, even at high concentrations (8 µL/well) with cell viability maintained at > 70%, providing a safe basis for *in vivo* applications. Simultaneously, an *in vivo* study found that after intravenous injection, MIL-88A nanoparticles are primarily captured by the reticuloendothelial system of the liver and spleen, and are gradually hydrolyzed and degraded into Fe ions and fumaric acid ligands. These degradation products are safely metabolized through a homeostatic mechanism, with no significant toxicity, confirming the metabolic safety of the carrier system 136.

Then, these nanoparticles were coated with EVs derived from HeLa cells, significantly enhancing their cellular uptake and therapeutic efficacy. The EV coating enabled controlled, intracellular drug release, as verified by fluorescence release experiments using a membrane-impermeable calcein model. The results demonstrated that this EV-MOF system prevented premature drug release and improved the transmission and release of chemotherapeutic agents within tumor cells. Although some studies have indicated that delayed release of the encapsulated drug in the body may reduce its anti-cancer activity and raise the half maximal inhibitory concentration (IC50) value for cancer cells. This phenomenon could be explained by the slower rate at which the nanoparticles release chemotherapy drugs, which prevents the drug from reaching sufficient concentrations quickly enough thereby delaying the response to cytotoxic effects 137. Nevertheless, other studies have suggested that free drugs exhibit greater cytotoxicity in the short term, while their rapid release may limit their sustained action within tumor cells. In contrast, sustained-release drugs, through sustained release and nuclear targeting, can interfere with tumor cell proliferation over a longer period, inhibiting the growth of rapidly dividing tumor cells 138. This slow-release mechanism compensates for the delayed toxicity response in the short term and provides a more lasting effect for tumor therapy by prolonging the duration of drug action. Therefore, the system leverages endogenous exosome pathways and nanocarrier degradation to enhance drug delivery while maintaining cell viability, making it a highly effective platform for cancer treatment.

#### 4.2.2 Protein therapy

**Figure 7:**
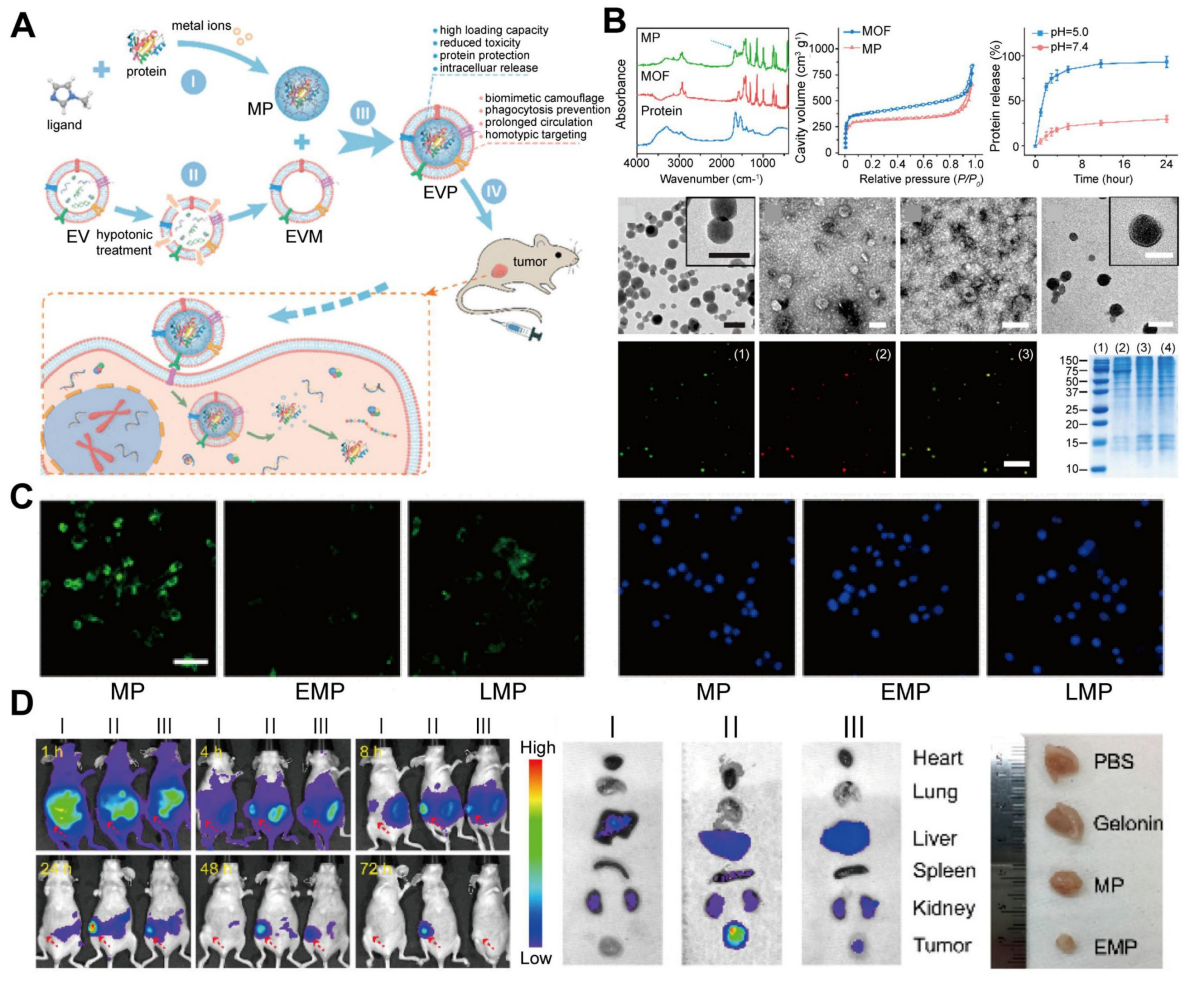
Protein therapy.** A**. Schematic for bionic EMP nanoparticle preparation and delivery. The MOF protein (MP) nanoparticles encapsulate the protein, and the extracellular vesicle membrane (EVM) further encloses it to form EMP nanoparticles for protein delivery. **B**. Assembly and characterization of biomimetic nanoparticles: Fourier transform infrared (FTIR) spectroscopy and N₂ adsorption-desorption isotherms confirmed the protein assembly in MP. The release efficiency of the guest protein was higher at pH 5.0. TEM images exhibited the MP, EV, EVM, and EMP morphologies (scale bar, 100 nm). Confocal microscopy revealed green fluorescence for encapsulated protein in MP and red fluorescence for the EVM (scale bar, 2 μm). Sodium dodecyl sulfate-polyacrylamide gel electrophoresis (SDS-PAGE) revealed the protein composition of (1) markers, (2) EVs, (3) EVM, and (4) bionic nanoparticles without loading cargo proteins. **C**. Laser scanning fluorescence microscopy of RAW264.7 cells incubated with MP, EMP, and liposomal-enveloped MP (LMP) nanoparticles for 2 h (nucleus: blue, nanoparticles: green; scale bar, 50 μm). EVM significantly reduced the internalization of the nanoparticles. **D**. Through systemic administration, EMP nanoparticles increased gelonin accumulation in tumors and inhibited growth. (I) Gelonin, (II) EMP, and (III) MP. Adapted with permission from 139, copyright 2018, American Chemical Society.

Protein therapy, a highly promising treatment approach, faces numerous challenges. Proteases easily degrade therapeutic proteins, significantly reducing their bioavailability and therapeutic efficacy. Additionally, a key challenge is the selective delivery of therapeutic proteins to target tissues while avoiding off-target effects in non-target tissues. Even if proteins are successfully delivered to target cells, achieving controlled release after intracellular internalization remains challenging, because it may cause excessively high or low intracellular drug concentrations, affecting therapeutic efficacy and increasing potential toxicity. To address these issues, Gong Cheng *et al*. 139 developed a biomimetic nanosystem using EM-ZIF-8 for protein delivery (**Figure 7**). In this system, proteins are encapsulated within pH-responsive MOF nanoparticles, protecting them from degradation and clearance while enabling efficient delivery to target cells. Zn^2+^ and 2-methylimidazole self-assemble to form a ZIF-8 framework that cages therapeutic proteins, such as gelonin, an rRNA-disrupting cancer therapeutic protein. This structure achieves high protein loading efficiency (~94%) and enables the controlled release of proteins in the acidic environment of endosomes and lysosomes. Additionally, coating the nanoparticles with EM helps them evade phagocytosis and prolongs their circulation time, enhancing their targeting of specific tumor cells 140,141.

*In vitro* and *in vivo* studies demonstrated that EM-coated MOF-protein nanoparticles protected protein cargos, reduced uptake by mononuclear phagocytic systems, and effectively delivered to tumors. This approach has the potential to enhance the efficacy of treatment by improving the delivery and protection of therapeutic proteins.

#### 4.2.3 Gene therapy

MicroRNAs (miRNAs) are non-coding RNAs with a length of 18-25 nucleotides that regulate gene expression and play critical roles in processes, including cell survival, proliferation, and tumor growth 142. Aberrant miRNA expression is closely associated with cancer progression, making miRNA-based therapies a promising approach in oncology 143,144. For instance, miR-34a has demonstrated potential in treating breast cancer by inhibiting silencing information regulator 1 expression 145,146. However, miRNAs face challenges, including poor stability, short half-life, and non-specific biodistribution, limiting their therapeutic efficacy 147.

To address these limitations, ZIF-8, a biocompatible MOF, has been used for miRNA delivery 148. Its porous structure and pH sensitivity allow it to encapsulate miRNAs and release them into the acidic environment of tumor cells, promoting endosomal escape and cytosolic delivery 149,150. However, the ability of ZIF-8 to non-specifically adsorb biomolecules in the bloodstream can lead to immune clearance, thereby reducing its effectiveness 151.

**Figure 8:**
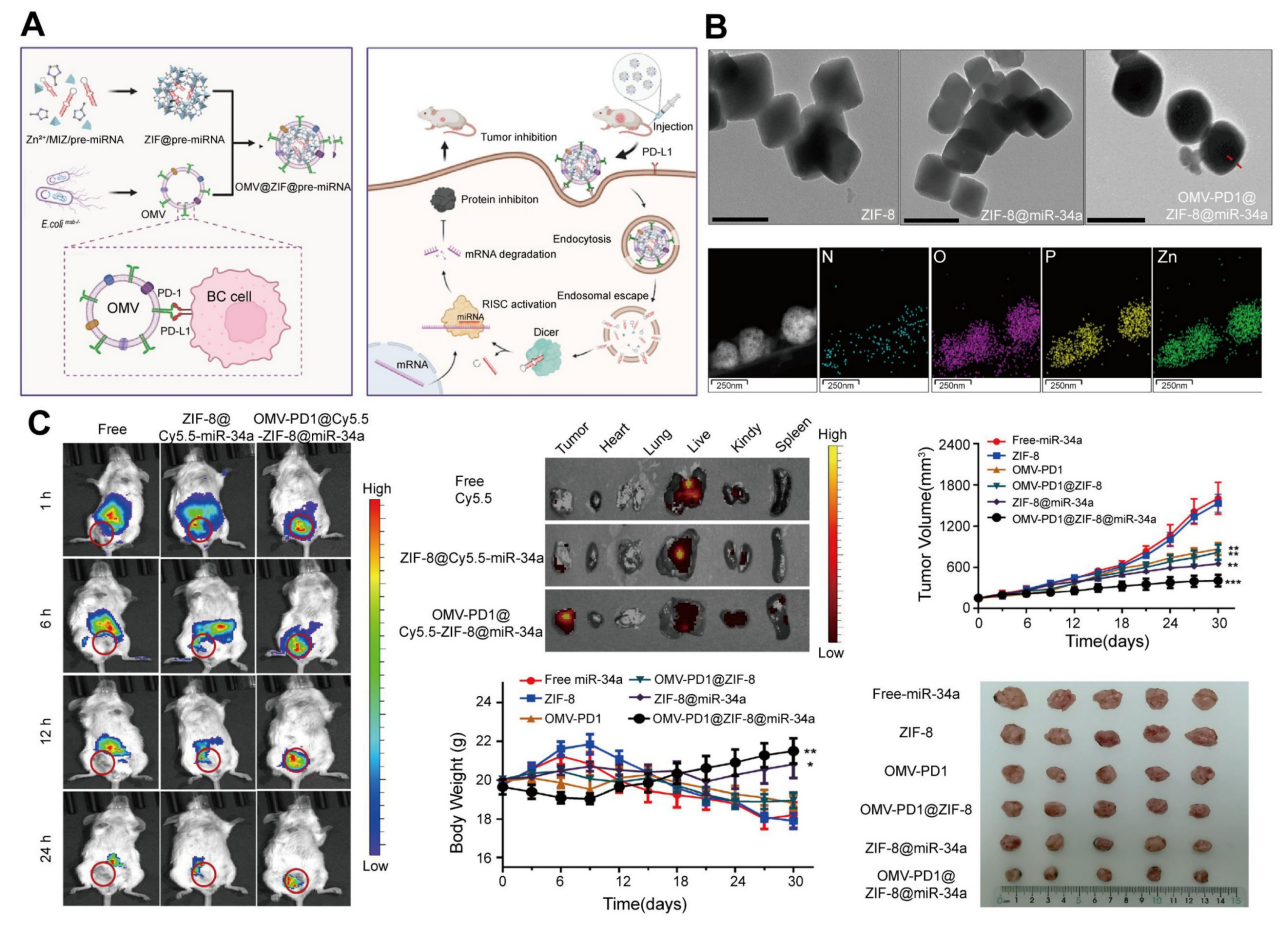
Gene therapy.** A**. Schematic of the preparation and targeted delivery of the OMV-PD1@ZIF-8@miRNA nanodelivery system and its therapeutic effect. **B**. TEM images of ZIF-8, ZIF-8@miR-34a, OMV-PD1@ZIF-8@miR-34a, and elemental mapping of OMV-PD1@ZIF-8@miR-34a (scale bar, 200 nm); arrows indicate OMV membranes. **C**. *In vivo* tumor-targeting and antitumor effects. The OMV-PD1@ZIF-8@Cy5.5-miR-34a exhibited rapid tumor accumulation, and enhanced tumor targeting, whereas the control group of free Cy5.5 and ZIF-8@Cy5.5-miR-34a preferentially accumulated in the liver and kidneys. The treatment group also exhibited significant inhibition of tumor growth and weight gain. Adapted with permission from 154, copyright 2023, Elsevier B.V.

A solution to this challenge involves coating ZIF-8 nanoparticles with 20-300 nm outer membrane vesicles (OMVs) derived from gram-negative bacteria 152. OMVs enhance the biocompatibility, targeting specificity, and immune evasion of the system while enabling synergistic miRNA and immunotherapy effects 153. Cui *et al*. 154 developed an OMV-coated ZIF-8 nanodelivery system for miRNA therapy. This platform efficiently delivered miR-34a to tumor cells, while OMVs displaying programmed death receptor 1 (PD1) on their surface provided targeted tumor recognition and immune activation. This combined effect enhances miRNA delivery and immune responses, leading to improved therapeutic outcomes in breast cancer. The OMV-ZIF-8 system represents a powerful tool for miRNA-based cancer therapies, offering enhanced stability, targeted delivery, and potential for synergistic immunotherapy (**Figure 8**).

#### 4.2.4 Immunization therapy

Ascorbic acid (AA)-mediated T cell-dependent therapy has gained attention in tumor immunology due to its potential to enhance immune responses against tumors 155. However, the rapid clearance of AA from the bloodstream and its limited efficacy in "cold" tumors, which lack immune cell infiltration, present significant challenges 156,157. Integrating AA with immunogenic cell death (ICD) inducers, such as bortezomib (BTZ), offers a strategy to stimulate immune response effectively 158,159. Nonetheless, achieving tumor-targeted delivery and controlled release of BTZ and AA requires an advanced delivery system capable of high payload stability, immune evasion, and selective release within the TME.

**Figure 9:**
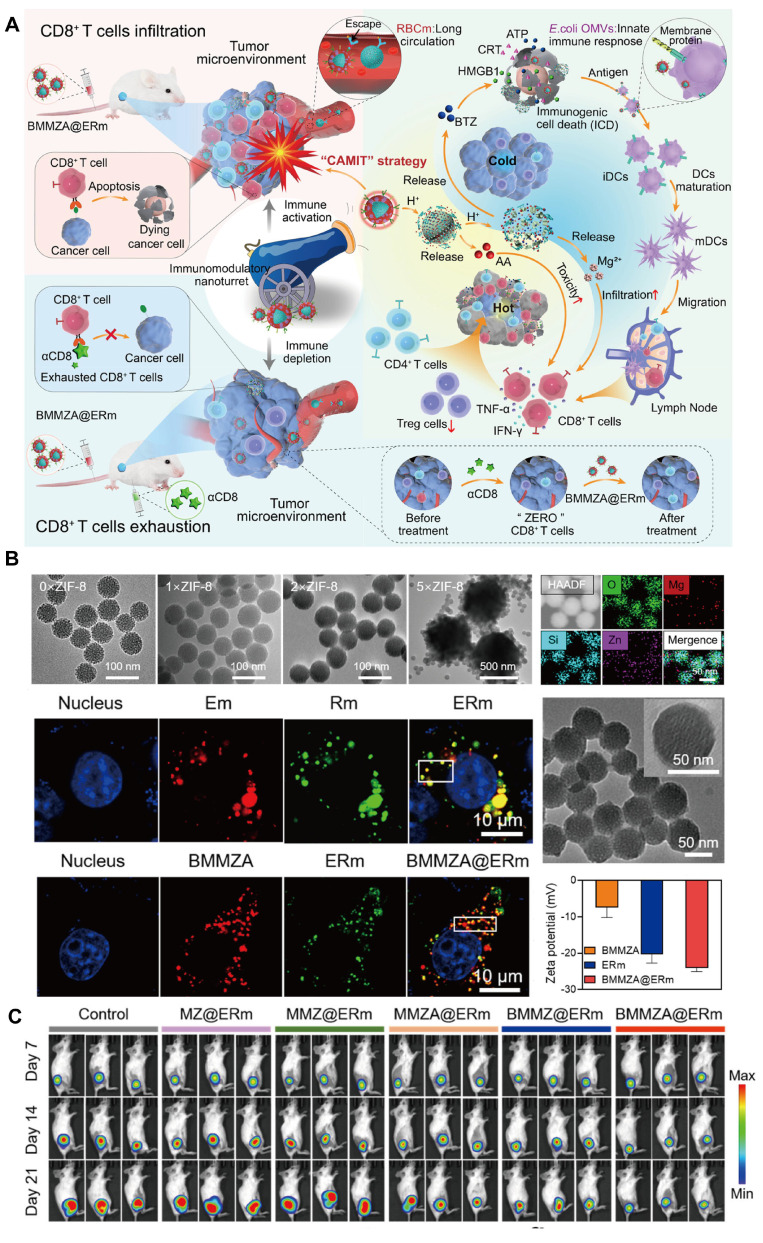
Immunization therapy.** A**. Schematic of an immunomodulatory nano-turret based on biodegradable nanocarriers camouflaged with hybrid *Escherichia coli* OMV-ERm, for chemotherapy-assisted ascorbic acid-mediated immunotherapy (CAMIT). **B**. TEM images of MMS@ZIF-8 synthesized with varied feeding concentrations of ZIF-8 precursors; element mapping of MMS@ZIF-8; the confocal laser scanning microscopy images of Em, Rm, hybrid ERm and BMMZA, ERm, BMMZA@ERm; TEM of BMMZA@ERm; and zeta potentials of BMMZA, ERm, and BMMZA@ERm. **C**. *In vivo* tumor therapy. Bioluminescence imaging revealed the BMMZA@ERm-treated mice demonstrated the strongest tumor inhibition. Adapted with permission from 160, copyright 2024, American Chemical Society.

To address these challenges, Yao *et al*. 160 developed a biomimetic nanocarrier, BMMZA@ERm, designed for the co-delivery of AA and BTZ (**Figure 9**). This system employs magnesium-doped mesoporous silica (MMS) nanocarriers with AA immobilized within a ZIF-MOF matrix, and MOF served as a pH-sensitive gate for BTZ-loaded MMS (BMMS). The nanocarrier was further camouflaged with a hybrid membrane derived from *Escherichia coli* OMVs and erythrocyte membranes (ERm), extending circulation time and enhancing immune compatibility. The MOF gate degrades in the acidic TME, enabling controlled and sequential release of AA and BTZ. This cascade facilitates potent ICD induction via BTZ, which promotes dendritic cell maturation and T-cell infiltration with the immunomodulatory effects of OMVs and Mg^2+^. Subsequently, AA subsequently activates T cells, converting "cold" tumors into "hot" ones with significant anti-tumor and antimetastatic effects.

This innovative AA-based immunotherapy platform demonstrates a T cell-dependent mechanism, as confirmed by CD8-deficient models, highlighting the potential of the system in personalized cancer immunotherapy. The BMMZA@ERm platform underscores the promise of integrating MOFs, ICD inducers, and AA in a multifaceted nanocarrier, offering new avenues for immunotherapy and overcoming immunological barriers within the TME.

#### 4.2.5 Sonodynamic therapy

Sonodynamic therapy uses low-intensity ultrasound to generate reactive oxygen species (ROS) at tumor sites, offering a non-invasive treatment with high tissue penetration 161. Despite these advantages, the accumulation of sonosensitizers in tumors is frequently hindered by biological barriers in the TME 162. Furthermore, many sonosensitizers, such as porphyrins and pyrroles, suffer from short half-lives and poor targeting, limiting their effectiveness in sonodynamic therapy 163,164.

To overcome these challenges, Zhang *et al*. 25 constructed an EV-coated MOF system for targeted delivery of sonosensitizers. In this approach, ZIF-8 nanoparticles were loaded with the sonosensitizer Ce6 and coated with OMVs derived from *Escherichia coli* MG1655. This coating enhanced the tumor-targeting ability and immune activation of the nanoplatform. *In vivo* studies using a mouse model of breast cancer demonstrated that this OMV-modified MOF system improved sonosensitizer accumulation in tumors, triggered effective immune responses in the TME, and enhanced the therapeutic efficacy of sonodynamic therapy. This platform represents a promising direction for future sonodynamic therapy applications in cancer therapy.

### 4.3 Treatment of rheumatoid arthritis (RA)

RA is a chronic inflammatory disease driven by dysregulated immune responses that cause joint destruction and systemic comorbidities 165. Conventional therapies, including non-steroidal anti-inflammatory drugs, glucocorticoids, and disease-modifying antirheumatic drugs, are frequently associated with severe side effects, such as osteoporosis, liver and kidney toxicity, and heightened infection risks 166,167. These limitations necessitate the development of advanced therapies to improve drug efficacy and minimize adverse effects.

To address these challenges, Liu *et al*. 34 designed a biomimetic nanoplatform by encapsulating TNFi within ZIF-8 nanoparticles, coated with membranes from M_2_NV. This AINUT platform protected TNFi from protease degradation and enhanced its targeted delivery to inflamed joints.* In vivo* studies have demonstrated that AINUT effectively reprogrammed inflammatory macrophages into anti-inflammatory subtypes and preferentially accumulated in the acidic RA joint environment, leading to significant therapeutic benefits.

Additionally, Wang *et al.* 106 developed a dexamethasone (Dex)-loaded ZIF-8 nanoplatform coated with macrophage-derived microvesicles (MVs) to improve Dex bioavailability. The resulting MV/Dex/ZIF-8 system prolonged drug circulation, enhanced stability, and allowed sustained release at inflamed joints, reducing the need for repeated dosing and minimizing side effects.

Furthermore, Wang *et al.* 168 developed a similar strategy to deliver methotrexate (MTX), a common RA treatment. They developed MV/MTX@ZIF-8 by encapsulating MTX in ZIF-8 nanoparticles and coating them with MVs, which targeted inflamed joints by overexpressing folic acid receptors on macrophages. This system enhanced drug accumulation in arthritic tissues, reduced hepatotoxicity, and improved overall therapeutic outcomes.

### 4.4 Treatment of ulcerative colitis (UC)

UC is a chronic inflammatory bowel disease characterized by recurrent inflammation and bleeding that profoundly affects a patient's quality of life 169. The global incidence and prevalence of UC have risen dramatically, with approximately five million cases reported worldwide as of 2023 170. Current therapies, including aminosalicylates, corticosteroids, and biological treatments such as anti-TNF agents 171, offer limited efficacy due to high dosage requirements, toxicity, and systemic side effects 172. Consequently, there is an urgent need for targeted therapies with high specificity and minimal systemic impact.

To address this therapeutic gap dilemma, Cui *et al*. 173 developed a novel TNF-α siRNA delivery platform (EVs@ZIF-8@siRNA) targeting UC. This design uses ZIF-8 to encapsulate and stabilize TNF-α siRNA, providing a robust delivery vehicle. To enhance targeting and bioavailability, the ZIF-8 nanocarrier was coated with ginger-derived EVs, which improved the acid stability in the gastrointestinal tract and promoted colon-specific targeting. Furthermore, 6-shogaol, an EV component of ginger, exerts anti-inflammatory effects, creating a synergistic interaction with TNF-α siRNA that amplifies therapeutic efficacy. This system also supports intestinal barrier integrity and modulates gut microbiota, fostering an environment conducive to mucosal healing and inflammation control in UC models.

### 4.5 Wound healing

Wound healing, particularly for chronic wounds, oral ulcers, and hypertrophic scars, is a major healthcare challenge 174. The increasing global incidence of diabetes, obesity, and aging populations has exacerbated the prevalence of chronic wounds, such as diabetic ulcers, resulting in high healthcare costs 175. In addition, more than 25% of the global population has experienced or is experiencing oral ulcers, seriously affecting the quality of life of patients 176. Similarly, hypertrophic scars caused by burns, trauma, or surgery frequently cause severe physical discomfort and psychological distress 177.

Mesenchymal stromal cell-derived EVs (MSC-EVs) have demonstrated great promise in promoting wound healing due to their involvement in angiogenesis, immunomodulation, and tissue regeneration 178,179. MSC-EVs are rich in miRNAs and cytokines that facilitate these processes 180; however, their rapid degradation and short lifespan limit their efficacy 181.

**Figure 10:**
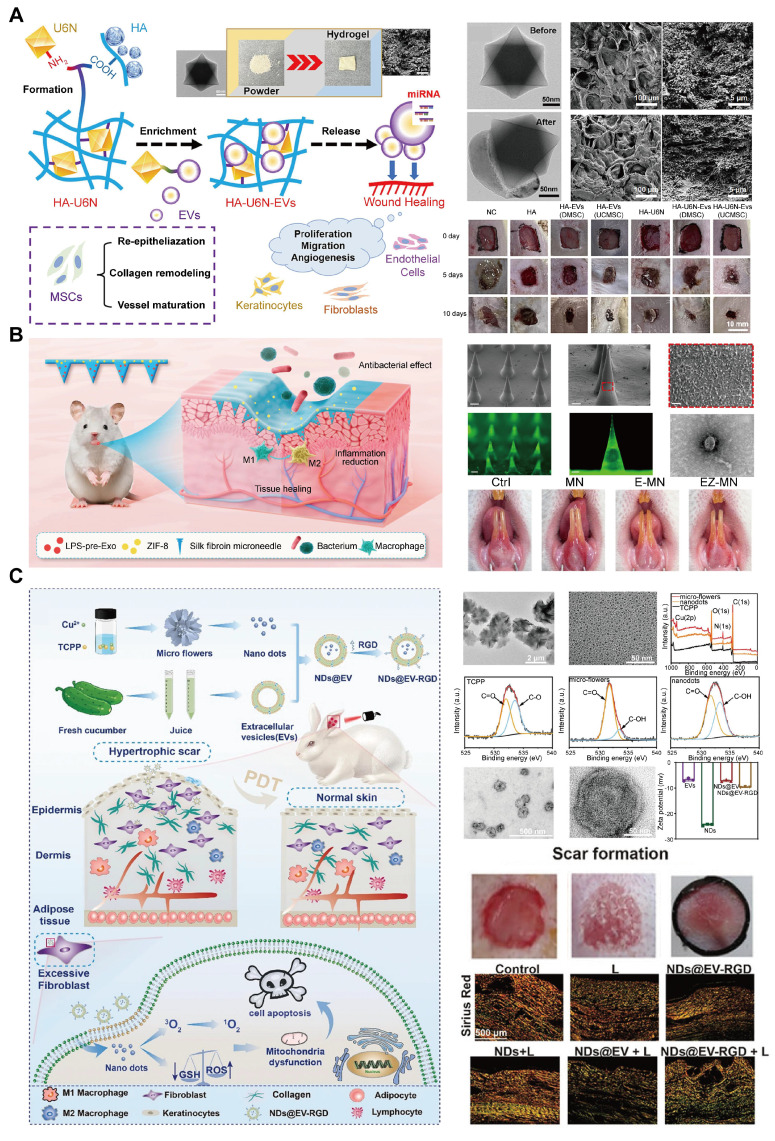
Wound healing.** A**. Schematic and characterization of MOF-based hydrogels (HA-U6N-EVs) for chronic wound healing. TEM images of MOFs and MOF-adsorbed EVs, SEM images of HA-U6N hydrogels before and after swelling, and wound closure progression in diabetic rats at 0, 5, and 10 days. MSC-EV-treated rats exhibited significant wound healing by day 5, whereas controls healed slowly. **B**. Schematic and characterization of MNs loaded with LPS-pre-Exos and ZIF-8 treating for oral ulcers. SEM images of normal MNs and images of MNs with green fluorescent substance added, along with TEM images of LPS-pre-Exos. Representative images displayed near-complete ulcer healing in the EZ-MN group with red mucosa, while the control group displayed slower healing. **C**. Schematic and characterization of NDs@EV-RGD nanoparticles for PDT-mediated hypertrophic scar treatment. TEM images of Cu-MOF microflowers, Cu-MOF NDs, EVs, and NDs@EV-RGD, along with the X-ray photoelectron spectroscopy (XPS) of TCPP, Cu-MOFs microflowers and Cu-MOFs NDs, and zeta potential measurements of EVs, NDs, NDs@EV, and NDs@EV-RGD. The efficacy of the treatment was demonstrated through scar imaging and three-color staining of the treated scars. (A) Adapted with permission from 182, copyright 2024, Elsevier B.V. (B) Adapted with permission from 183, copyright 2024, American Chemical Society. (C) Adapted with permission from 186, copyright 2024, Wiley-VCH GmbH.

To address this issue, MOF-based delivery systems have been developed to protect EVs, prolong their release, and enhance their therapeutic effects. Wang *et al*. 182 developed a UiO-66-NH2-based hydrogel that effectively adsorbs MSC-EVs via strong Zr-O-P coordination bonds. This hydrogel protects the EVs and enables their sustained release at wound sites, thereby accelerating tissue regeneration. In diabetic wound models, the hydrogel system significantly enhanced wound closure and tissue repair (**Figure 10A**).

Ge *et al*. 183 developed a silk fibroin microneedle patch to treat oral ulcers that included lipopolysaccharide (LPS)-preconditioned bone marrow mesenchymal stem cells and their secreted exosomes (LPS-pre-Exos) alongside ZIF-8 for the treatment of oral ulcers (**Figure 10B**). This microneedle (MN) patch design facilitates painless, minimally invasive, and targeted transdermal exosome delivery, enabling sustained release of LPS-pre-Exos and promoting macrophage polarization toward a pro-healing phenotype. Furthermore, the controlled release of Zn^2+^ from ZIF-8 contributes to the anti-inflammatory and antimicrobial properties of the patch, fostering an environment conducive to rapid tissue regeneration and ulcer healing. This innovative MN patch offers a promising localized, and sustained-release approach for enhancing mucosal wound healing.

Photodynamic therapy (PDT) has gained attention for treating hypertrophic scars due to its ability to soften scar tissue and reduce pain 184. However, poor transdermal delivery of photosensitizers frequently limits the PDT efficacy 185. To overcome this, Kong *et al*. 186 developed a copper-based MOF nanoplatform, functionalized with EVs and arginine-glycine-aspartic acid (RGD) peptides for targeted delivery. This system enhances skin penetration and ROS generation under near-infrared light, promoting fibroblast apoptosis and collagen remodeling in hypertrophic scar tissues 187. EVs can overcome the barrier of the dense stratum corneum (SC) due to their high affinity for SC, which is primarily composed of lipids, as well as their superdeformation ability, allowing them to penetrate the skin surface layer 188,189. These studies highlight the potential of EV-MOF hybrid systems in addressing the challenges of chronic wounds and hypertrophic scars, providing innovative solutions for effective and sustained wound healing therapies (**Figure 10C**).

### 4.6 Bone regeneration

Reconstructing large bone defects caused by trauma, inflammation, or tumors is a significant challenge in orthopedics 190. In addition to promoting osteogenesis, early vascularization is critical for supplying nutrients and removing waste during bone formation 191-193. Accordingly, regulating osteogenesis and angiogenesis is essential for bone regeneration.

EVs derived from hADSCs have demonstrated promise in enhancing bone regeneration 194. However, their rapid degradation and short half-life limit their efficacy 195,196. To address this, researchers have used MOFs as scaffolds, which offer tunable porosity, biocompatibility, and controlled release properties. For example, ZIF-8-modified hydrogels have been demonstrated to promote angiogenesis and osteogenesis 197.

Mg^2+^ has also proven beneficial in bone regeneration by enhancing autophagic activity and promoting calcium deposition, essential for bone growth 198,199. Moreover, Mg^2+^ exhibits angiogenic and anti-inflammatory effects 200,201, making it an ideal component in bone tissue engineering. Kang *et al*. 47 developed a PLGA/Mg-GA MOF scaffold, incorporating hADSCs-EVs, Mg^2+^, and gallic acid (GA) to enhance osteogenesis, angiogenesis, and anti-inflammatory reduction. *In vitro* experiments have demonstrated that this matrix can significantly promote osteogenesis of hBMSCs and angiogenesis of HUVECs. The slowly released hADSC-EVs provided a stable growth environment for the cells and accelerated bone regeneration. *In vivo* studies have revealed that this scaffold accelerates bone remodeling and promotes successful osseointegration, indicating its promising clinical application prospects.

Cu^2+^ has also been recognized for its role in promoting osteogenesis and angiogenesis 202-204. Xu *et al*. 205 combined Cu-based MOFs with EVs from human bone mesenchymal stem cells to create a scaffold that provided sustained release of bioactive ions and EVs, upregulated the expression of osteogenic-related proteins in hBMSCs, promoted angiogenesis and formation, and significantly enhanced bone regeneration in a rat model. These findings highlight the potential of EV-MOF scaffolds as powerful tools for promoting vascularized bone regeneration, offering a promising strategy for the treating of large bone defects.

### 4.7 Anti-infection

The rise of multidrug-resistant (MDR) and extensively drug-resistant (XDR) bacterial strains has raised serious global health concerns 206,207. Conventional antibiotics are becoming increasingly ineffective, prompting the need for novel antimicrobial strategies 208. MOFs and EVs have emerged as promising platforms for combating infections by providing new approaches for targeted delivery, enhanced drug efficacy, and controlled release 209.

MOFs can encapsulate and protect antimicrobial agents, prevent degradation and enhance their delivery to infected sites 210. Furthermore, the ability of MOFs to bypass bacterial efflux pumps and resist bacterial enzymatic degradation makes them a potent solution against MDR pathogens 211. Combining EVs with MOFs further enhances targeting efficiency and antimicrobial effects, enabling the precise delivery of antimicrobial agents to infection sites 212. This synergy between MOFs and EVs opens up new possibilities for treating numerous infections, including biofilm-associated and systemic bacterial infections.

A notable example is the development of the ZnBq/Ce6@ZIF-8@OMV nanoplatform that integrates a Zn-based metal complex (ZnBq) with Ce6 as a photosensitizer and is encapsulated within ZIF-8 MOF nanoparticles 213. These nanoparticles were further coated with genetically engineered OMVs to enable precise targeting and enhanced antimicrobial action. The ZnBq/Ce6@ZIF-8@OMV system demonstrated exceptional efficacy in treating infections caused by *Acinetobacter baumannii*, a leading MDR pathogen in healthcare settings. The MOF component stabilizes and protects the antimicrobial agents, while the OMV coating ensures targeted delivery, allowing the nanoplatform to penetrate biofilms and effectively eliminate bacteria. This system also offers the added benefit of PDT, in which Ce6 is activated by light to generate ROS, further enhancing its bactericidal activity. The success of the ZnBq/Ce6@ZIF-8@OMV platform underscores the potential of EV-MOF hybrid systems as next-generation anti-infective therapies, that can address the limitations of current antibiotics and offer new hope against resistant infections.

In addition to traditional MOF-based antibacterial agents, porphyrin MOFs with conjugated systems and electrocatalytic properties have emerged as potent electrosensitizers in electrodynamic therapy (EDT) for bacterial infections. EDT, an innovative non-antibiotic approach, leverages an electric field to generate ROS and provides a particularly advantageous targeted bactericidal effect combating bacterial biofilms. Concurrently, plant-derived EVs are promising for neutralizing bacterial toxins due to their phospholipid membrane structure, which mimics host cell membranes and interacts with bacterial toxins. Based on this, Wang *et al*. 214 developed a bionic nano-sponge (MOF@EV) camouflaged with ginger-derived EVs to camouflage and functionalize with nano-porphyrin as an electrical sensitizer. This system effectively absorbs toxins and generates ROS under electrical stimulation, achieving potent anti-biofilm and antibacterial effects. In both *in vitro* and *in vivo* experiments, MOF@EV exhibited superior biocompatibility and effectively cleared *Staphylococcus aureus*-induced subcutaneous abscesses, demonstrating its potential in clinical anti-infective therapies.

Currently, biocatalytic therapy targeting bacterial pathogens has emerged as a novel therapeutic strategy for antimicrobial treatment 215. Studies have demonstrated that in the presence of nitric oxide synthase (iNOS), L-arginine undergoes biocatalysis to generate nitric oxide (NO) 216. NO can be toxic to bacteria by inhibiting bacterial DNA recombination and causing severe peroxidative stress in biofilms 217. Wan *et al*. 218 constructed a nanobiohybrid nanoreactor EV@ZIF, mimicking the natural biomineralization process to deposit a ZIF layer on the EV surface, thereby enhancing the reactor stability. Besides, EVs inherently possess iNOS activity that can catalytically generate NO, while the ZIF, as a photocatalyst, can absorb light energy under illumination to produce photogenerated electron-hole pairs. The photogenerated electrons transfer to the EVs, bind with NADP^+^, and promote its reduction to NADPH, a key coenzyme in the iNOS enzymatic reaction, thereby accelerating the conversion of NO. This enables EV@ZIF to efficiently generate NO under illumination, with a yield far exceeding that of unmodified EVs. The system exhibited strong bactericidal activity (~70%) and the ability to penetrate the biofilm in antibacterial experiments, significantly inhibited the inflammatory response of gingival tissue and promoted periodontal tissue repair in animal experiments, suggesting its potential for treating infectious diseases such as periodontitis.

Additionally, the challenges of implant-associated infections (IAIs), particularly for orthopedic implants, are increasingly pressing due to the global demand for effective, infection-resistant implants 219. IAIs are especially resistant to conventional treatments due to the anaerobic biofilms that form on implant surfaces, protecting bacterial cells from immune responses and reducing antibiotic efficacy 220. Therefore, non-surgical surface engineering is thus critical for mitigating IAIs by directly enhancing antibacterial efficacy at the implant surface while promoting bone regeneration 221. Li *et al*. 222 introduced a novel coating strategy that combines an ultrasonically-activated copper-based MOF (Cu-TCPP), probiotic-derived OMVs, hypoxia-induced antibiotic tinidazole (TNZ), and poly-tannic acid (pTA) to form an antimicrobial coating layer for titanium implants. Under ultrasonication, Cu-TCPP catalyzes the production of singlet oxygen (^1^O_2_) and generates a hypoxic environment to enhance the antibacterial effects of TNZ. Simultaneously, Cu (II) ions from Cu-TCPP were reduced to Cu (I), disrupting bacterial metabolism, thereby causing cell death. The OMVs layer modulates immune response by promoting macrophage polarization to an M_2_ phenotype, reducing inflammation, and enhancing osteogenic factor expression, thereby fostering an infection-resistant bone-regenerative microenvironment. This multifunctional coating displays promise for effective biofilm prevention, improved bone integration, and long-term protection against reinfection.

### 4.8 Analysis of signal path mechanism

The EV-MOF system has demonstrated therapeutic effects in various diseases, including inhibition of the proliferation and metastasis of tumor cells, alleviation of inflammation in RA, improvement of the intestinal microenvironment in UC, promotion of wound healing, and acceleration of bone tissue regeneration. However, current research lacks a systematic analysis of the regulatory mechanisms of core signaling pathways, limiting a comprehensive understanding of its mechanism of action and hindering the clinical translation of EV-MOF 223.

Research on RA, has already yielded EV-MOF studies that use omics technologies to explore signaling pathway mechanisms. The study employed a global RNA profiling approach to investigate primary M_1_ macrophages and M_1_ macrophages treated with M_2_NVs. The pathways altered after treatment with M_2_NVs were concentrated in the nuclear factor-kappa B (NF-κB), Toll-like receptors (TLRs), and mitogen-activated protein kinase (MAPK) signaling pathways, which play dominant roles in macrophage polarization. In M_1_ macrophages treated with M_2_NVs, the expression of anti-inflammatory regulatory genes (Foxp3, MapK11, and Gdf15) was upregulated, while the expression of pro-inflammatory genes was downregulated. M_2_NVs demonstrate a robust immunoregulatory capacity by modulating pathways related to macrophage polarization 34.

However, in other diseases, such as cancer, abnormal activation of signaling pathways, including phosphatidylinositol 3-kinase (PI3K)/protein kinase B (AKT) and extracellular signal-regulated kinase (ERK) is closely related to tumor malignancy 224. There is still a lack of conclusive evidence on whether and how the EV-MOF system achieves anti-cancer effects by regulating these key signaling pathways. Similarly, in UC, the regulatory mechanisms of signaling pathways involved in intestinal inflammation and repair, such as Wnt/β-catenin and transforming growth factor-β (TGF-β) pathways, require in-depth study 225. Further research into the regulatory mechanisms of signaling pathways, such as TGF pathway, and vascular endothelial growth factor (VEGF) signaling pathway, which involve cell proliferation, migration, and angiogenesis, is required for wound healing 226,227. In terms of bone regeneration, the regulatory mechanisms of signaling pathways, such as bone morphogenetic protein (BMP) and Wnt pathways, that control bone formation and resorption remain poorly understood 228.

Most current studies use only traditional molecular biology methods, such as WB, quantitative polymerase chain reaction (qPCR) to detect changes in the expression levels of a small number of key proteins or genes, making it difficult to fully understand the complex dynamic processes in signaling pathways. The limitations of this research method result in a limited understanding of the overall mechanism of the EV-MOF system in intracellular signal regulation 154.

To systematically address this knowledge gap, it is recommended that comprehensive multi-omics research be conducted. Proteomics techniques can be used to comprehensively analyze the expression and modification profiles of hundreds or thousands of proteins within cells, potentially revealing the modulatory effects of the EV-MOF system on multiple nodes of signaling pathways. Dynamic changes in the phosphorylation levels of key proteins in signaling pathways can be monitored using quantitative proteomics combined with the analysis of phosphorylation modifications, thereby inferring the regulatory role of the EV-MOF system 229. Transcriptomic techniques can comprehensively analyze changes in gene expression within cells, helping to trace upstream regulatory events in signaling pathways and when integrated with proteomic data, can provide a complete regulatory network from the transcriptional to protein level 230. Additionally, metabolomics research can reveal whether the EV-MOF system indirectly affects the activity of signaling pathways by regulating intracellular metabolomic pathways 231. Integrating these multi-omics data may provide a new perspective to understand the role and mechanism of the EV-MOF system in different diseases 232.

In-depth mechanistic research is important to guide the rational design of next-generation EV-MOF systems. Only by clarifying the regulatory mechanisms of EV-MOF systems in signaling pathways can these systems be precisely optimized based on the pathological characteristics and signaling pathway abnormalities of different diseases. For example, if it is found that a certain EV-MOF system can inhibit the PI3K-AKT signaling pathway in tumor cells, its inhibitory effect can be further enhanced by chemically modifying MOF materials or genetically engineering EV components, thereby improving cancer treatment efficacy 233,234. Similarly, mechanistic research based on corresponding signaling pathways for RA, UC, wound healing, bone regeneration, and anti-infection may provide a theoretical foundation for developing EV-MOF systems with improved bioactivity.

In summary, integrated systems research helps to reveal the mechanisms by which the EV-MOF system regulates intracellular signaling pathways, laying a solid theoretical foundation for the rational design of next-generation EV-MOF systems. This may promote the clinical translation of EV-MOF systems treating various diseases, opening up new avenues for future precision medicine and regenerative medicine research.

## 5. Challenges and Prospects

The synergistic application of EVs and MOFs presents transformative potential in nanomedicine; however, several key challenges must be addressed for clinical translation.

(1) Large-scale production and material integration: Scalability, large-scale production, and EV-MOF integration are the major barriers. Current traditional separation technologies for EVs, such as ultracentrifugation, have low yields and significant batch-to-batch quality variability, limiting the stable supply of therapeutic-grade exosomes 235. To overcome this, advances in bioreactors and microfluidics are critical for the continuous, large-scale production of EVs 236,237. The traditional MOFs synthesis process requires high temperatures, high pressures, or organic solvent environments and are highly sensitive to minor changes in synthetic conditions, frequently resulting in poor batch consistency of nanostructures, high laboratory costs, and commercialization barriers. Optimizing synthetic conditions, such as low-temperature continuous flow processes and green solvent alternatives, and tuning crystal structures, such as ZIF-8 and MIL-101, can reduce costs 238. Furthermore, the methods used to couple EVs with MOFs, such as ultrasound, extrusion, or co-incubation, vary in efficiency and consistency 239. Standardizing these methods is necessary to ensure high binding efficiency and structural integrity across different EV-MOF systems. Evaluating these approaches using metrics, such as loading capacity and purity, is crucial for optimizing therapeutic outcomes. Furthermore, artificial or engineered EVs can be designed to optimize their compatibility with MOFs 240,241, providing a more standardized system for clinical applications.

(2) Stability and functional integrity: EVs are susceptible to degradation, limiting their therapeutic window 242. Developing cryopreservation and lyophilization techniques that maintain the structural and functional integrity of EV-MOF systems is essential 243,244. Currently, cryoprotectants, such as trehalose have been developed to stabilize the structure of EVs 245. Simultaneously, novel EV preservation methods have been developed, such as the non-destructive encapsulation of EV via ZIF-8, which, while developing as well as new EV preservation methods, provides a new perspective on the combination form of the EV-MOF system by achieving MOF synthesis and EV encapsulation in one step 39. Additionally, the consistency of biological functions must be ensured for functional integrity by real-time monitoring of key parameters of EVs, such as particle size and surface markers 246. Moreover, the design of MOF scaffolds must ensure controlled degradation and sustained release of EV cargo while maintaining bioactivity over longer periods.

(3) Safety and immunogenicity: Although EVs improve the biocompatibility of MOFs, safety concerns arise when using EVs derived from cancer cells, which may promote tumor growth if not carefully engineered 247. Alternatives such as non-cancer cell-derived EVs or engineered can mitigate this risk, while maintaining effective targeting and immune evasion 248. Establishing safe EVs sources, such as mesenchymal stem cells (MSCs) or macrophages, may be pivotal in future applications. Although the dissociation of the structure and release of metal ions in MOFs can result in the release of drugs and therapeutic factors by drug carriers reaching targets in the body, excessive and highly toxic metal ions and ligands can cause immunocompatibility issues 249,250. Choosing endogenous metal ions, such as Zn^2+^ and Fe^3+^ and natural ligands, such as cyclodextrin, can reduce immunotoxicity due to their specific metabolic pathways and biodegradability 251,252.

(4) Mechanistic understanding and preclinical research: A deeper understanding of EV-MOF interactions *in vivo* is necessary. Key questions regarding biodistribution, biotransformation, and cellular uptake remain unresolved 253. Developing tracers to track EV-MOF in real time, along with extensive preclinical studies in relevant disease models, helps elucidate their mechanism of action and optimize therapeutic delivery. Furthermore, comprehensive pharmacokinetic and pharmacodynamic studies are vital for assessing the effectiveness and safety of EV-MOF systems before clinical trials 254. Yao *et al*. 160 reported that the circulation half-life of BMMZA@ERm (11.78 h) was significantly prolonged compared to BMMZA (3.42 h), and it could be completely excreted through urine and feces within 48 h.

(5) Quality monitoring and regulatory review: The clinical transformation of EV-MOF composite products must overcome the dual regulatory challenges posed by agencies such as the FDA for biological products and nanomaterials 255. Although the Pharmaceuticals and Medical Devices Agency of Japan's Scientific Committee issued the first official guideline for EV treatment products by an official regulatory body 256, there is still a global lack of a specialized regulatory framework for MOF and EV-MOF composite products. Their review needs to integrate existing standards for biological products, such as traceability of EV sources and batch-to-batch consistency requirements, and nanomaterials standards, such as MOF cytotoxicity thresholds and degradation kinetics 257,258, and, using artificial intelligence (AI)-driven tools and global platforms such as the International Council for Harmonisation of Technical Requirements for Pharmaceuticals for Human Use (ICH) platform, promote the development of adaptive regulatory models, to achieve cross-innovation transformation in regenerative medicine and nanotechnology 259,260.

**Figure 11:**
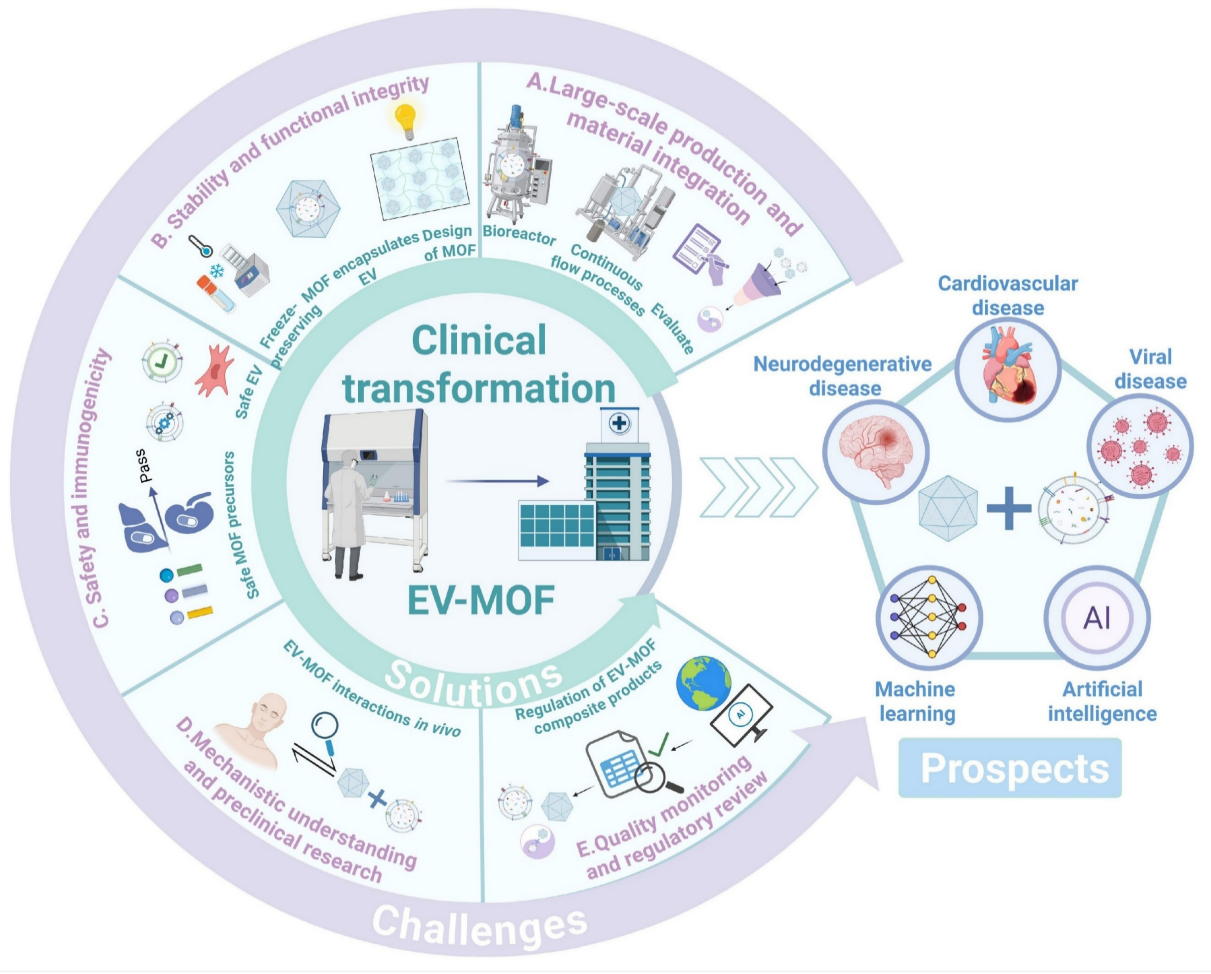
Challenges in the clinical transformation of EV-MOF, corresponding solutions, and prospects for EV-MOF systems. The circular outer ring and adjacent purple text depicts the challenges in the clinical transformation of EV-MOF. The inner circle and adjacent green font, along with the graphics inside the sector, display the solutions for the corresponding challenges. The pentagon on the right presents the prospects for the EV-MOF system. Created using BioRender.com.

The EV-MOF platform holds promise for theranostics by combining diagnostics and therapeutics in a single system for future clinical applications. Beyond cancer, they have potential applications in cardiovascular, neurodegenerative, and viral diseases, where precise targeting and controlled drug release are critical. As AI and machine learning are integrated into the design and optimization of EV-MOF systems 261,262, these platforms may become highly personalized, further advancing precision medicine (**Figure 11**).

## 6. Conclusion

The synergistic application of EVs and MOFs has introduced a transformative approach to nanomedicine, offering advanced solutions for diagnostics and therapeutics. By leveraging the unique properties of MOFs, such as high porosity and tunable release, alongside the inherent biocompatibility and targeting capabilities of EVs, EV-MOF systems have demonstrated significant potential in enhancing disease detection, particularly through improved sensitivity in biomarker identification and imaging technologies. In the therapeutic context, EV-MOF platforms offer efficient drug delivery systems that ensure controlled release and reduced toxicity, as evidenced by their promising applications in cancer treatment, RA, and bone regeneration. However, challenges remain in scaling production, ensuring long-term stability, and understanding the *in vivo* mechanisms of EV-MOF interactions. Addressing these issues through continued research is crucial for realizing the full clinical potential of EV-MOF systems. As nanomedicine advances toward more personalized and precise treatments, EV-MOF systems are poised to play a crucial role, offering innovative pathways for overcoming the current limitations in diagnosis and therapy. Their integration into clinical practice can advance this field by offering safer and more effective therapeutic strategies and enhancing early disease detection.

## Figures and Tables

**Table 1:** Performance comparison of EV-MOF systems and traditional nanomedicine carriers.

Comparison parameters	EV-MOF	Liposomes	Polymer nanoparticles	Inorganic nanoparticles	References
Drug loading efficiency	High (high MOF porosity)	Medium (depends on phospholipid membrane)	Medium (dependent on adsorption or coupling)	High (part of the materials are mesoporous structures with large pore sizes)	62,63
*In vivo* stability	Medium (some MOFs are prone to hydrolysis; EV encapsulation can improve)	Medium (easily removable, PEGylation can improve)	Medium (biodegradability)	High (chemical inertness)	64-69
Immunogenicity	Low (CD47 and other membrane proteins of EV evade immune clearance)	Medium (PEG coating can extend circulation time but may induce anti-PEG antibodies)	Low (most have good immunogenicity; hydrophobic polymers have high immunogenicity)	High (directly induces immune responses)	71-73
Targeting specificity	High (passive targeting and natural homing)	Low (passive targeting)	Low (passive targeting)	Low (passive targeting)	74-76

**Table 2:** Summary of methods for combining EVs with MOFs or composite MOFs.

Convergence technology	Technical principle	Advantages	Disadvantages	References
Co-incubation	MOFs and EVs are incubated together at a specific temperature.	Simple operation; less structure and integrity damage to EVs.	Low loading efficiency; limited mass production.	101
*In situ* encapsulation	Through mixing EVs with MOF precursors, MOFs are formed *in situ* on the EV surface, resulting in the encapsulation of EVs.	High preparation efficiency; one-step sequential coating.	Possible EV bioactivity impairment.	39
Ultrasound	Ultrasonic energy acts on EVs to form transient channels, allowing MOFs to enter and form core-shell nanostructures.	High loading efficiency; less material loss.	More structure and integrity damage to EVs; limited mass production.	102
Extrusion	EVs and MOFs are extruded by porous membranes, and EVs are cracked and reassembled around the surface of MOFs to form core-shell nanostructures.	High loading efficiency.	More structure and integrity damage to EVs; limited mass production.	103
Microfluidic ultrasound	Microfluidics combined with ultrasound to cause batch EVs to rupture and recombine around MOFs to form core-shell nanostructures.	Streamlined operation; mass-production capability.	Specialized equipment requirement; elevated cost.	104
Extrusion after ultrasound	Ultrasonic energy and the mechanical force of extrusion cause the EVs to wrap on the surface of the MOFs.	Enhanced loading efficiency.	More structure and integrity damage to EVs; limited mass production.	105
Co-incubation after ultrasound	After ultrasonic treatment, when MOFs enter EVs, incubation at 37°C can restore the integrity of the EM.	Enhanced loading efficiency; increased restoration of EV structure and membrane integrity.	Limited mass production.	106

## Abbreviations

3D: three-dimensional
AA: ascorbic acid
AI: artificial intelligence
AKT: protein kinase B
ALF: artificial lysosomal fluid
ATP: adenosine triphosphate
Au: gold
BMMS: BTZ-loaded MMS
BMP: bone morphogenetic protein
BTZ: bortezomib
CAMIT: chemotherapy-assisted ascorbic acid-mediated immunotherapy
CD-MOFs: cyclodextrin-based MOFs
CT: computed tomography
Cu-TCPP: copper-based MOF
Dex: dexamethasone
DLS: dynamic light scattering
EDT: electrodynamic therapy
EM: EV membranes
ERK: extracellular signal-regulated kinase
EPR: enhanced permeability and retention
ERm: erythrocyte membranes
EVM: extracellular vesicle membrane
EV-MOF: extracellular vesicle-metal organic framework
EVs: extracellular vesicles
FDA: U.S. Food and Drug Administration
Fe: iron
FTIR: Fourier transform infrared
GA: gallic acid
hADSCs: human adipose-derived mesenchymal stem cells
IAIs: implant-associated infections
IC50: half maximal inhibitory concentration
ICD: immunogenic cell death
ICH: International Council for Harmonisation of Technical Requirements for Pharmaceuticals for Human Use
iNOS: nitric oxide synthase
LPS: lipopolysaccharide
LPS-pre-Exos: lipopolysaccharide-preconditioned bone marrow mesenchymal stem cells and their secreted exosomes
LMP: liposomal-enveloped MP
M_2_NV: M_2_ macrophage-derived vesicle
mAuNSs: Au nanostars
MB: methylene blue
MDR: multidrug-resistant
Mg: magnesium
miRNAs: microRNAs
MMS: magnesium-doped mesoporous silica
MN: microneedles
MOFs: metal-organic frameworks
MP: MOF-protein
MRI: magnetic resonance imaging
MSCs: mesenchymal stem cells
MSC-EVs: mesenchymal stromal cells-derived EVs
MTT: 3- (4,5)-dimethylthiahiazo (-z-y1)-3,5-di- phenytetrazoliumromide
MTX: methotrexate
MVs: macrophage-derived microvesicles
NGPs: nanolecular particles
NO: nitric oxide
NTA: nanoparticle tracking analysis
^1^O_2_: singlet oxygen
OMVs: outer membrane vesicles
PD1: programmed death receptor 1
PDT: photodynamic therapy
PEG: polyethylene glycol
PI3K: phosphatidylinositol 3-kinase
pTA: poly-tannic acid
qPCR: quantitative polymerase chain reaction
RA: rheumatoid arthritis
RhB: rhodamine B
ROS: reactive oxygen species
SDS-PAGE: sodium dodecyl sulfate-polyacrylamide gel electrophoresis
SEM: scanning electron microscopy
TEM: transmission electron microscopy
tExo: tumor-derived exosomes
TGF-β: transforming growth factor-β
Ti: titanium
TME: tumor microenvironment
TNF-α: tumor necrosis factor-α
TNFi: TNF-α inhibitors
TNZ: tinidazole
UC: ulcerative colitis
VEGF: vascular endothelial growth factor
WB: western blotting
XDR: extensively drug-resistant
XPS: X-ray photoelectron spectroscopy
ZIF-8: zeolite imidazolate frameworks-8
Zn: zinc
Zr: zirconium
